# Recent Advances in Multi-Atom Catalysts for Sustainable Energy Applications

**DOI:** 10.3390/molecules30132818

**Published:** 2025-06-30

**Authors:** Qing Wang, Bo Cheng, Shichang Cai, Xiaoxiao Li, Di Lu, Naying Zhang, Chaoqun Chen, Hanlu Zhang, Yagang Feng, Lei Duan, Shaoyong Qin, Zihan Meng

**Affiliations:** 1 School of Material Science and Engineering, Henan University of Technology, Zhengzhou 450001, China; 15222926921@163.com (Q.W.); bo_cheng@haut.edu.cn (B.C.); 18749900977@139.com (X.L.); 18300696424@163.com (D.L.); 18238827157@163.com (N.Z.); 15538906081@163.com (C.C.); hanlu_zhang@haut.edu.cn (H.Z.); yagangfeng@163.com (Y.F.); lei_duan@haut.edu.cn (L.D.); 2 National Energy Key Laboratory for New Hydrogen-Ammonia Energy Technologies, Foshan Xianhu Laboratory, Foshan 528200, China; 3 School of Materials Science and Engineering, Xi’an University of Technology, Xi’an 710048, China; qinshaoyong@xaut.edu.cn

**Keywords:** electrochemical reactions, single-atom catalysts, multi-atom catalysts, sustainable energy

## Abstract

Single-atom catalysts characterized by their novel electronic configurations and exceptional atomic utilization efficiency have emerged as potential alternatives to costly noble metal catalysts, garnering extensive research attention in various electrocatalytic fields. However, the inherent characteristics of single metal centers constrain their further application in catalyzing multi-electron reactions. In contrast, multi-atom catalysts (MACs), particularly dual-atom catalysts (DACs), possess multiple active metal sites that can significantly enhance catalytic performance through synergistic effects. This review summarizes recent developments in multi-atom catalysts, focusing on synthesis methods, design strategies, and the correlation between interatomic synergy and catalytic efficiency. Furthermore, we discuss their applications in key electrochemical reactions, including the hydrogen evolution reaction, oxygen reduction reaction, and oxygen evolution reaction. Finally, we outline the opportunities and challenges in this field to guide the development of high-efficiency catalysts for sustainable energy conversion applications.

## 1. Introduction

**Figure 1:**
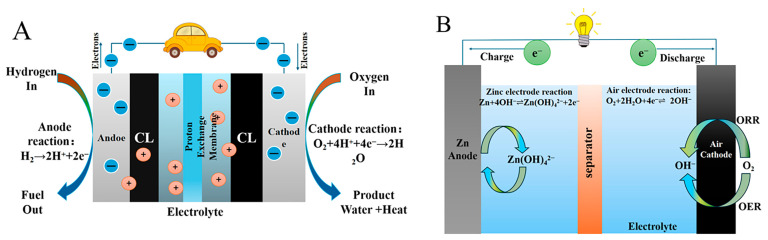
Schematic illustration of PEMFC (**A**) and ZABs (**B**).

The global commitment to carbon neutrality has exposed the limitations of conventional fossil fuel dependency, manifesting in critical challenges of resource shortages and environmental contamination. The development of green energy has become an urgent necessity to address the current energy crisis and the degradation of the environment [1,2,3]. Among several clean energy strategies, the effective operation of systems such as proton exchange membrane fuel cells (PEMFCs) [4,5,6,7,8], zinc–air batteries (ZABs) [9,10,11,12], and H_2_ production via water electrolysis critically relies on electrocatalysts that accelerate three fundamental electrochemical reactions: the oxygen reduction reaction (ORR) [13,14,15,16], oxygen evolution reaction (OER) [17,18,19,20,21,22], and hydrogen evolution reaction (HER) [23,24,25,26,27,28,29]. Taking PEMFCs as an example, their high energy conversion efficiency [30] (40~60%), low-temperature rapid start-up capability (<80 °C), and zero carbon emissions make them one of the cornerstone technologies in clean energy conversion. A typical PEMFC system is composed of the anode catalyst layer (ACL) [31], cathode catalyst layer (CCL) [32], proton exchange membrane (PEM) [33], gas diffusion layer (GDL) [34], and bipolar plate (BP) [35], as schematically represented in Figure 1A. During their operation, molecular hydrogen (H_2_) is electrochemically oxidized at the ACL through the hydrogen oxidation reaction (HOR) [36,37] (HOR: H_2_ → 2H^+^ + 2e^−^), generating proton carriers and free electrons. The liberated electrons are transferred through an external circuit to perform electrical work, while the protons migrate through the hydrated PEM (e.g., Nafion^®^ membrane) to the cathode compartment. Simultaneously, molecular oxygen (O_2_) introduced at the CCL participates in the ORR (O_2_ + 4H^+^ + 4e^−^ → 2H_2_O), which synergistically combines protons, electrons, and O_2_ to generate waste heat and water. However, the ORR process is inherently constrained by its multi-step proton-coupled electron transfer (PCET) mechanism, introducing substantial kinetic barriers that dominate polarization losses and ultimately restrict the fuel cell’s power density and operational efficiency. Similarly, ZABs have emerged as highly promising contenders for future energy storage systems owing to their environmental benignity, low cost, and exceptional theoretical energy density [38] (1086 Wh kg^−1^), in addition to the ORR process that takes place during the discharge process at the air electrode side (Figure 1B). Both PEMFCs and ZABs necessitate high-performance cathode catalysts to mitigate the activation overpotential (*η*) of ORR, which is a fundamental step for achieving enhanced efficiency in electrochemical energy storage devices. Platinum-group metal (PGM) catalysts have demonstrated benchmark electrocatalytic performance for ORR owing to their optimal binding energy with O-containing intermediates (*O, *OH, *OOH). A Pt/C catalyst (Pt/C-nR) with an ultra-high loading capacity of 85 wt% was synthesized by You et al. [39] using a multi-step reduction technique. They proposed that the multi-layered stacked Pt nanoparticle structure significantly enhances Pt utilization, thereby boosting the efficiency of direct methanol fuel cells (DMFC). The stacked structure formed via multi-step reduction effectively decreases the proportion of Pt particles buried in carbon micropores, increasing the electrochemical surface area (ECSA) from 60.2 m^2^/g Pt in the single-step reduction (Pt/C-1R) to 65.3 m^2^/g Pt, with utilization rates rising from 68% to 90%. This material exhibited a high ORR efficiency with a mass activity that was 1.7 times greater compared to that of a single-step reduction (0.85 V vs. RHE) and achieved a 21% increase in DMFC single-cell power density (49.4 vs. 40.9 mW/cm^2^).

In addition, water electrolysis holds significant importance for enabling large-scale green hydrogen economies through sustainable H_2_ production [40,41,42]. This technology involves the electrochemical decomposition of H_2_O to generate H_2_ and O_2_, with its core mechanism relying on electrical energy to drive the OER and the HER processes at the anode and cathode, respectively. The compatibility of this technology with intermittent green energy sources (e.g., wind power and photovoltaic) makes it particularly suited for synergistic grid-scale energy storage and decarbonized hydrogen synthesis. Notably, similar to the charge cycle in zinc–air batteries, the OER also governs the anode processes during water electrolyzer operations. For HER catalysis, advanced electrocatalysts must optimize the Volmer–Heyrovsky–Tafel pathways by reducing the free energy of hydrogen adsorption (ΔG_H), thereby minimizing activation overpotential. While PGMs exhibit near-ideal ΔG_H values (~0 eV) and benchmark HER activity, their scarcity and associated costs hinder widespread implementation in gigawatt-scale electrolyzers. The OER process presents greater fundamental challenges due to its kinetically sluggish four-electron pathway (4OH^−^ → O_2_ + 2H_2_O + 4e^−^), requiring catalysts to stabilize high-valent metal-oxo intermediates (e.g., *O, *OOH) while withstanding corrosive oxidative environments. State-of-the-art OER catalysts predominantly employ iridium/ruthenium oxides (IrO_2_, RuO_2_), yet their industrial adoption remains constrained by material durability issues and unsustainable noble metal requirements.

**Figure 2:**
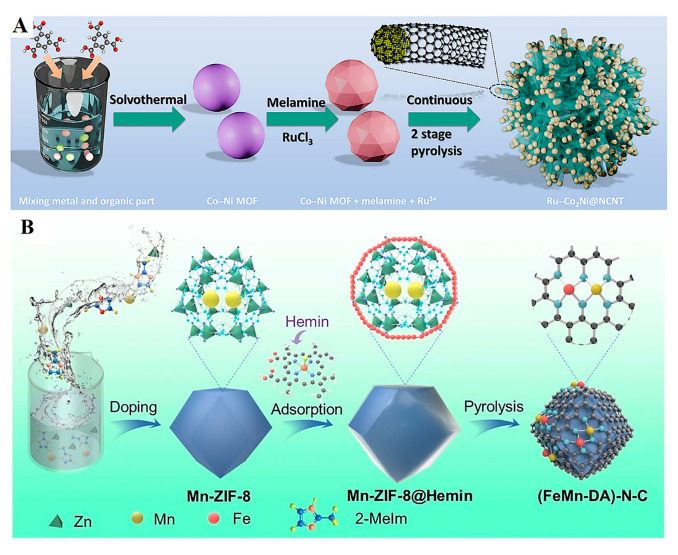
(**A**) A schematic diagram illustrating the preparation process of Ru/Co_2_/Ni @NCNT. Reprinted with permission from Ref. [43]. Copyright © 2025 Wiley-VCH GmbH. (**B**) Heme is represented by a red sphere in the magnified image of Mn-ZIF-8 @ Hemin. Reprinted with permission from Ref. [51]. Copyright © 2024, The Author(s).

Nowadays, state-of-the-art electrocatalytic systems for ORR, OER, and HER predominantly depend on noble metal-based catalysts such as Ru, Ir, and Pt. However, the exorbitant costs and scarcity of these materials impede their industrial-scale deployment. To close the gap between the fundamental research and commercial application of renewable energy technologies, the development of cost-effective catalysts with noble metal-comparable activity has emerged as a critical pathway. Current research focuses on two strategic approaches: (1) Maximizing noble metal atom efficiency through nanostructure engineering, as exemplified by core–shell-structured Ru/Co_2_/Ni nanoparticles encapsulated within N-doped carbon nanotubes [43] (Figure 2A). This architecture incorporates multiple synergistic mechanisms, including Ru active-site optimization, carbon nanotube density modulation, lattice strain effects, and interfacial water dissociation enhancement. (2) Another approach is designing non-noble metal catalysts to circumvent intrinsic cost limitations. For instance, while Pt-based catalysts dominate ORR catalysis in fuel cells, their prohibitive expense (>40% of total system costs) drives urgent demand for alternatives [44,45,46]. Consequently, engineering highly active, low-cost, and durable non-noble metal catalysts represent critical research development for sustainable energy conversion.

The conceptual foundation of single-atom catalysis was established through the seminal work of Zhang Tao’s group in 2011 [47], which pioneered the “single-atom catalysts” (SACs) concept and synthesized a platinum-based SAC (Pt_1_/FeO_x_) via atomic anchoring technology. This catalyst demonstrated exceptional CO oxidation activity, achieving a fivefold enhancement over conventional nanoparticle counterparts. This breakthrough not only established theoretical groundwork for SACs but also propelled rapid advancement in the field. Subsequent developments have yielded SAC synthesis methodologies, including atomic layer deposition and impregnation–pyrolysis techniques, with certain systems (e.g., Fe-N-C ORR catalysts) reaching kilogram-scale production. However, SACs encounter challenges such as the inadequate cooperative adsorption of intermediates in complex reactions and atom aggregation at elevated temperatures. To address these challenges, research has evolved toward dual-atom catalysts (DACs) and multi-atom catalysts (MACs). The construction of dual-metal active centers (e.g., Fe-Co [48,49,50] pairs) optimizes intermediate adsorption energy distribution, achieving 30–50 mV positive shifts in ORR half-wave potentials (*E*_1/2_). Concurrently, non-noble metal systems and nitrogen-doped carbon composites exhibit noble metal-comparable performance across wide pH ranges through interface engineering and defect modulation (Figure 2B) [51].

Critical challenges remain in balancing activity–stability tradeoffs and developing scalable synthesis protocols for non-precious catalysts. Future research directions should focus on (1) dynamic mechanism elucidation: employing operando characterization tools (e.g., X-ray absorption fine structure spectroscopy) to probe reaction interfaces; (2) manufacturing innovation: designing high-throughput synthesis platforms for precise atomic-scale control; and (3) rational catalyst design: establishing structure–activity relationships to guide performance-oriented material development.

**Figure 3:**
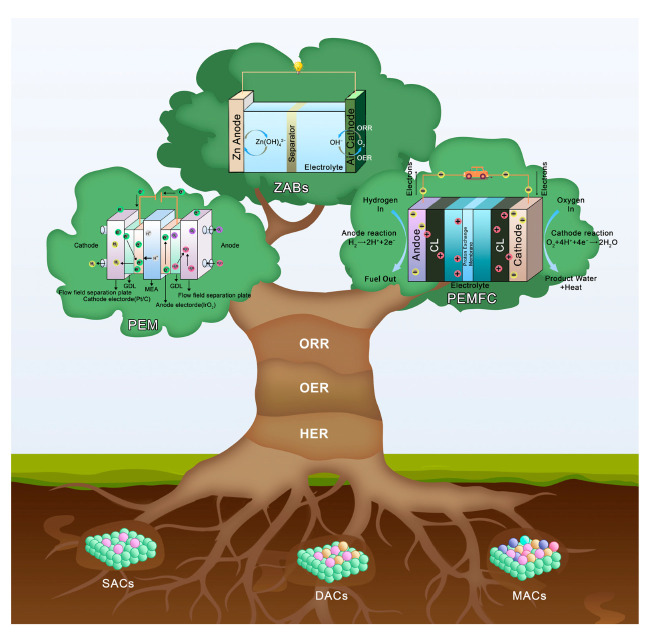
A schematic illustration of SACs, DACs, and MACs and their applications in ZABs, PEMFC, and water electrolysis.

In summary, this article systematically reviews the mechanisms of electrochemical reactions, specifically ORR, OER, and HER. We critically evaluate an overview of recent advancements in dual/multi-atom catalyst research, delving into advanced preparation methods, rational design strategies, interatomic synergistic effects, and underlying reaction mechanisms (Figure 3). Finally, this article summarizes and forecasts the challenges and opportunities in multi-atom catalyst materials implementation for next-generation energy conversion technologies.

## 2. The Reaction Mechanisms of ORR, HER, and OER

### 2.1. ORR

**Figure 4:**
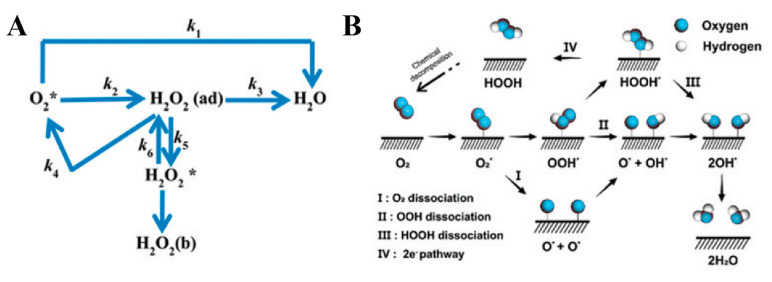
(**A**,**B**) A schematic diagram of the ORR mechanism. * denotes the active site. Reprinted with permission from Ref. [52]. Copyright © 2020, American Chemical Society.

The ORR represents a fundamental cathodic process in energy conversion systems such as metal–air batteries and fuel cells, which involve a multi-electron transfer pathway for oxygen reduction (O_2_ + 4H^+^ + 4e^−^ → 2H_2_O) (Figure 4) [52]. The reaction kinetics and mechanistic pathways are critically governed by the local electronic configuration of catalytically active regions and the surrounding reaction microenvironment, which has led to persistent debates regarding the identification of the rate-determining step (RDS).

In alkaline environments, the ORR predominantly occurs via a four-electron transfer route. This process may occur either via direct outer-sphere electron transfer to molecular oxygen or through an adsorption–displacement mechanism involving hydroxide species (OH^−^) at catalytic active sites. Conversely, under acidic conditions such as those encountered in proton exchange membrane fuel cells, the initial chemisorption of molecular oxygen emerges as the crucial rate-limiting step (RLS). This step requires the precise optimization of oxygen binding energy in accordance with the Sabatier principle [53,54] (ΔG_O_2_ ≈ 0.8–1.2 eV) to achieve an optimal balance between adsorption activation and intermediate desorption. Excessive oxygen adsorption strength leads to the accumulation of OOH* intermediates, leading to an increase in the value of *η* by approximately 0.3 V. Conversely, insufficient adsorption significantly compromises the efficiency of O-O bond cleavage, as exemplified by the 60% reduction in catalytic activity observed for the Pt(111) facet compared to the Pt(100) facet. Liu [55] significantly improved the oxygen adsorption performance by constructing a catalyst with an asymmetric active site: FeN_3_S_1_. The special structure of the FeN_3_S_1_ C meso-catalyst induces an enhancement in the electron density surrounding the Fe atom and reduces the d-band center of the Fe atom. This change in electronic structure optimizes the adsorption energy between the Fe atom and the OH* intermediate, thereby improving the inherent activity of the catalyst.

This pH-dependent mechanistic divergence highlights the critical structure sensitivity of catalytic active sites. For instance, Fe-N_4_ coordination sites promote adsorbed O_2_ dissociation in acidic ORR through axial water molecule coordination, whereas Co-N_2_O_2_ configurations lower the activation energy barrier in alkaline media via hydroxyl-assisted PCET mechanisms. However, the lack of comprehensive in situ characterization data regarding the dynamic reconstruction processes at the electrode–electrolyte interface presents a major challenge for the precise mechanistic elucidation of ORR pathways. According to the typical multi-electron ORR model established by Wroblowa et al. [56], molecular oxygen can undergo either a direct four-electron (4e^−^) reduction to H_2_O or a sequential two-electron (2e^−^) reduction to H_2_O_2_, as shown in Table 1 and Table 2. The direct 4e^−^ pathway demonstrates superior energy conversion efficiency and inherent resistance to H_2_O_2_-induced catalyst poisoning. On most non-noble metal catalysts, ORR proceeds through competing parallel pathways, with 4e^−^ transfer selectivity serving as a crucial performance metric for catalytic materials. The 4e^−^ pathway is particularly advantageous for fuel cell applications due to its higher thermodynamic efficiency and complete oxygen reduction. Conversely, the 2e^−^ pathway generates reactive peroxide species that induce three critical drawbacks: (i) catalyst surface passivation through intermediate adsorption, (ii) the corrosion of carbon-based support materials, and (iii) substantial energy efficiency losses. Although peroxide products could theoretically undergo further 2e^−^ reduction to water, a practical catalyst design must prioritize the suppression of the 2e^−^ pathway to maximize system performance and durability.

**Figure 5:**
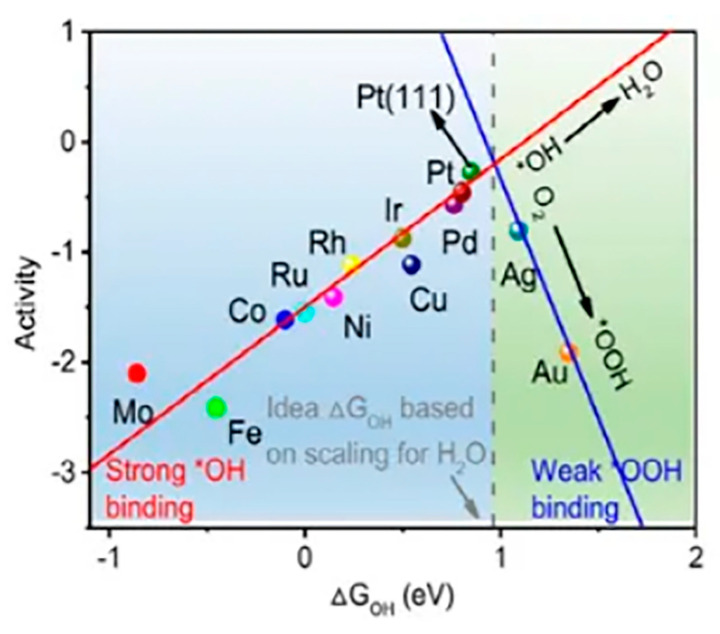
Volcanic curve of ORR activity. * denotes the active site. Reprinted with permission from Ref. [57]. Copyright © 2021 Science Press and Dalian Institute of Chemical Physics, Chinese Academy of Sciences, Published by Elsevier B.V. and Science Press. All rights reserved.

Nørskov [57] and co-workers established oxygen-containing species adsorption energy as a key descriptor for constructing the volcano-shaped activity curve of the oxygen reduction reaction. As illustrated in Figure 5, the ORR volcano graph is governed by two distinct potential-determining steps (PDS), each corresponding to the two branches of the curve: (i) The left branch (strong adsorption regime), where OH desorption (OH → H_2_O) constitutes the RLS. (ii) The right branch (weak adsorption regime) where *OOH formation (O_2_ → *OOH) serves as the PDS.

According to the existing data and content of the blue region in Figure 5 [58], it can be clearly concluded that the desorption of the product is more difficult due to the strong adsorption of the intermediate product (*OH → H_2_O, PDS). At the same time, the surface coverage of the catalytic sites used to adsorb O_2_ also becomes lower, and the ORR process is hindered for a series of reasons. In contrast, according to the green region on the right branch, it can be identified that the protonation of O_2_ (O_2_ → *OOH, PDS) and the further cleavage of the O-O bonds are limited by weak binding, which may reduce the kinetics and even initiate the 2e^−^ reaction.

### 2.2. HER

**Figure 6:**
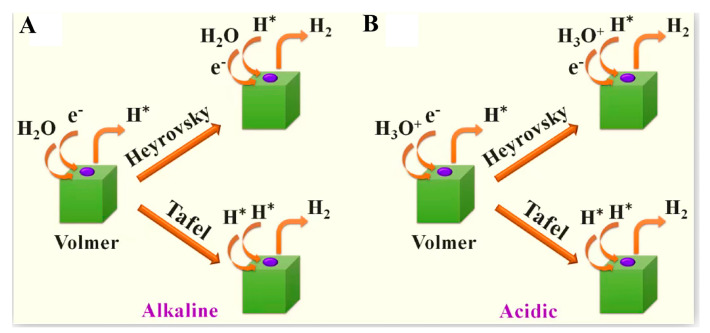
(**A**,**B**) HER mechanism diagram. * denotes the active site. Reprinted with permission from Ref. [59]. Copyright © 2022 Hydrogen Energy Publications LLC. Published by Elsevier Ltd. All rights reserved.

The HER process is a crucial cathodic half-reaction in water electrolysis for H_2_ production and electrochemical synthesis. Its fundamental principle involves proton reduction and hydrogen molecule formation (2H^+^ + 2e^−^ → H_2_ or 2H_2_O + 2e^−^ → H_2_ + 2OH^−^) (Figure 6) [59], and the reaction efficiency is constrained by proton adsorption/desorption kinetics and the binding energy of hydrogen intermediates.

**Figure 7:**
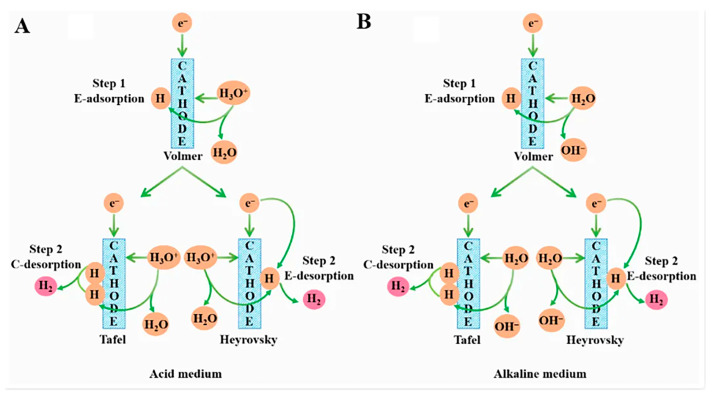
HER mechanisms (Volmer-Tafel and Volmer-Heyrovsky mechanisms) under acidic (**A**) and alkaline (**B**) conditions. Reprinted with permission from Ref. [60]. Copyright © 2022, American Chemical Society.

As shown in Table 3, HER can be classified into the Volmer–Tafel–Heyrovsky mechanism in acidic conditions (the formation and recombination of adsorbed H*) and the water-splitting–reduction coupling pathway in alkaline conditions (requiring a higher energy barrier to be overcome) based on the difference in the acidity or alkalinity of the reaction medium. The specific process can be summarized into two steps. The initial step is the Volmer reaction, in which H* is formed by reacting with H_3_O^+^ in acidic media and with H_2_O within alkaline media. The subsequent step is the Tafel or Heyrovsky process (Figure 7) [60]. This is the RDS that is intrinsically linked to the electronic structure of the catalyst. For example, Pt-group metals exhibit near-ideal ΔGH* (~0 eV), enabling efficient Tafel recombination, while transition metal catalysts typically require higher energy inputs for hydrogen desorption, often favoring Heyrovsky pathways.

Comprehensive analysis establishes how platinum-based catalysts demonstrate superior catalytic performance in HER, which is attributable to their near-optimal hydrogen adsorption free energy. While Pt-based materials (e.g., Pt/C) remain the industrial benchmark electrocatalysts, their prohibitive cost and scarcity have motivated extensive research into cost-effective alternatives, including non-noble metal catalysts (e.g., MoS_2_, Ni/Co phosphides) and atomically dispersed SACs. HER plays a critical role in large-scale green H_2_ production (coupled with anode OER), the efficient energy supply for hydrogen fuel cells, and electrochemical ammonia synthesis. The design of active sites and the interface engineering optimization of the catalysts are key to improving energy conversion efficiency and reducing hydrogen production costs.

### 2.3. OER

The oxygen evolution reaction constitutes the fundamental anodic process in clean energy technologies such as metal–air batteries and water electrolyzers. The reaction mechanism involves four sequential PCET steps (4OH^−^ → O_2_ + 2H_2_O + 4e^−^), which are inherently kinetically sluggish due to the complex multi-electron oxidation pathway and high-energy intermediates. This necessitates high-performance catalysts to mitigate the substantial activation energy barrier and enhance reaction kinetics.

Recent studies have shown that transition-metal-based catalysts (e.g., Ni/Fe-layered double hydroxides and IrO_2_) reduce the reaction overpotential through the adsorbate evolution mechanism (AEM) or the lattice oxygen oxidation mechanism (LOM) [61,62]. The AEM path depends on the gradual desorption/adsorption of *OOH intermediates, while the LOM path demands the direct involvement of lattice oxygen in the development of O-O bonds. OER plays a critical part in the preparation of green H_2_ (coupled with a cathodic hydrogen evolution reaction), an electrochemical energy storage system, and a CO_2_ reduction coupling device. The optimization of its stability and catalytic efficiency is essential to enhance the comprehensive efficiency of energy conversion equipment.

**Figure 8:**
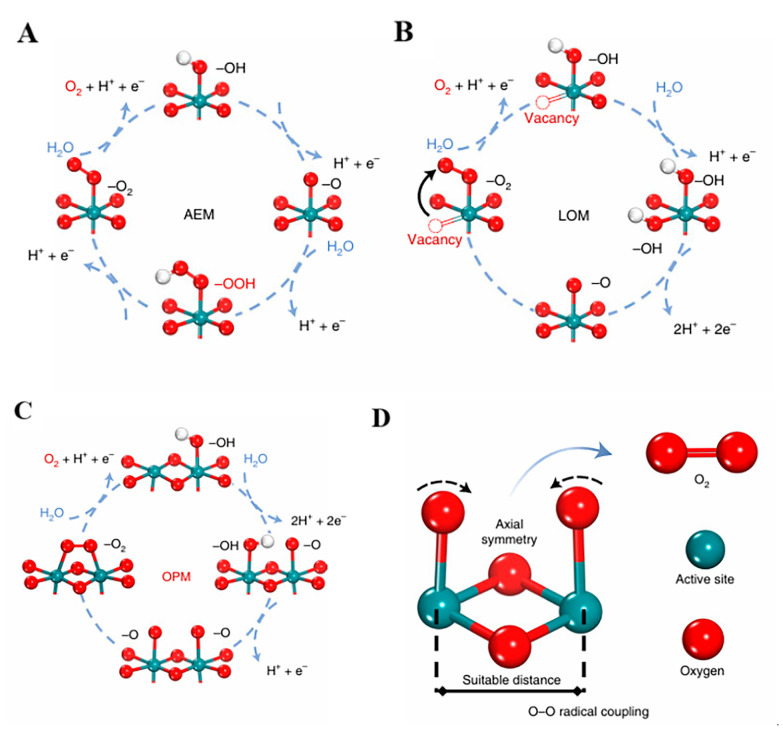
The schematic representation depicts the OER mechanisms in a heterogeneous Ru-based electrocatalyst. Simplified OER mechanisms are illustrated schematically. (**A**) AEM; (**B**) LOM; (**C**) OPM mechanism; and (**D**) a diagram illustrating O–O radical coupling mediated by symmetric dual active sites. Reprinted with permission from Ref. [63]. Copyright © 2021, The Author(s), under exclusive license to Springer Nature Limited.

The OER mechanism exhibits distinct reaction pathways within alkaline and acidic environments under the four-electron transfer mechanism. As illustrated in Figure 8 [63], the AEM involves the sequential adsorption/desorption of intermediate species through the OH_ads_ → O_ads_ → OOH_ads_ → O_2ads_ process.

**Figure 9:**
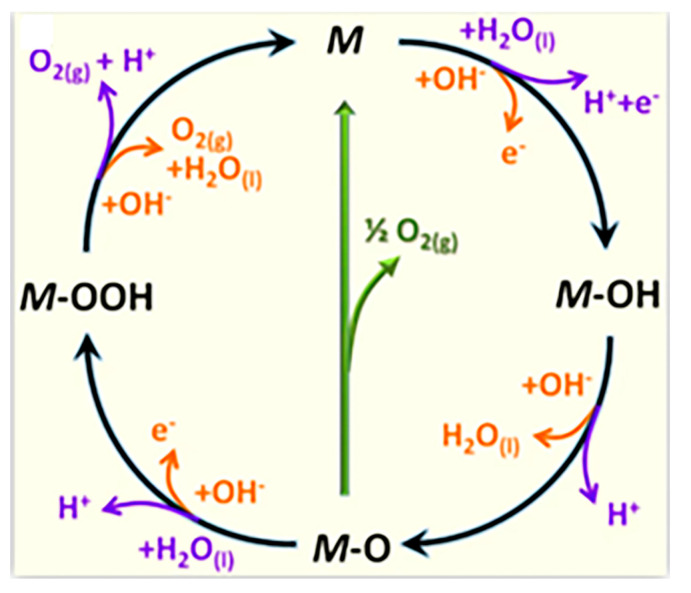
An OER mechanism schematic diagram illustrating the pathways. (The purple line represents the OER mechanism under acidic conditions; the orange line denotes the alkaline condition pathway; the black line indicates oxygen evolution involving the M-OOH intermediate formation; and the central green arrow signifies direct oxygen generation bypassing the M-OOH intermediate.). Reprinted with permission from Ref. [59]. Copyright © 2022 Hydrogen Energy Publications LLC. Published by Elsevier Ltd. All rights reserved.

As shown in Table 4, under acidic conditions, water molecules adsorb onto the catalyst surface and dissociate into adsorbed hydroxyl groups (OH*) and protons (H^+^), accompanied by electron release. Then, the adsorbed OH* undergoes further oxidation through electron transfer, forming adsorbed oxygen species (O*) with concurrent proton generation. Two adjacent O* intermediates combine to form molecular oxygen (O_2_), which subsequently desorbs from the catalytic surface. Throughout this process, protons migrate through the electrolyte solution while electrons migrate from the catalyst surface to the anode via the external circuit. Under alkaline conditions, hydroxide ions (OH^−^) initially adsorb onto catalytic active sites (OH*_ads_). The adsorbed OH*_ads_ loses an electron to form O intermediates, generating water molecules as byproducts. Two O* species combine to produce molecular oxygen (O_2_), which desorbs from the catalyst surface. This pathway necessitates the continuous regeneration of hydroxide ions through water dissociation and electron transfer processes in the electrolyte to sustain the reaction cycle (Figure 9) [59]. In summary, the reaction kinetics are fundamentally governed by the energy barriers associated with intermediate transformations, particularly the generation of OOH* species, which typically constitutes the RDS. This mechanistic understanding provides essential guidance for designing advanced electrocatalysts with optimized adsorption energies for key intermediates.

## 3. DACs

### 3.1. Noble Metal-Based DACs

Noble metal-based DACs have emerged as novel materials in energy catalysis, organic synthesis, and environmental remediation owing to their distinctive dual-metal synergy and atomically precise active site engineering (Table 5). Theoretical and experimental studies reveal that tailored dual-metal centers (e.g., Pt/Pd, Pt/Fe, and Ag/Pd) enable the optimization of intermediate adsorption-free energy profiles and a reduction in activation barriers, thereby improving both catalytic selectivity and activity. For instance, ordered Pt/Fe intermetallic compounds demonstrate exceptional ORR performance with a mass activity equal to 3.16 mA cm^−2^, which is attributed to the Fe-mediated electronic structure modulation of Pt and stabilization of lattice oxygen. Similarly, the Pd/Cu diatomic system achieves 95.3% propylene selectivity in propyne hydrogenation through balanced hydrogen activation and product desorption kinetics, exemplifying how reaction pathway control is enabled by dual-metal coordination environments. Furthermore, synergistic metal interactions enhance catalyst durability against poisoning and sintering, as evidenced by W_6_Co_7_ systems that suppress carbon deposition through high melting point characteristics while improving methane activation efficiency.

Despite these advantages, two critical challenges hinder their widespread implementation: (i) economic constraints from noble metal scarcity, particularly evident in platinum-based catalysts, and (ii) the thermodynamic instability of atomic dispersions, where metal atoms tend to migrate and agglomerate under operational conditions (elevated temperatures/reactive environments). Current stabilization strategies rely on advanced support engineering (nitrogen-doped carbons, metal–organic frameworks) and interfacial anchoring techniques. However, synthesis strategies such as microwave-assisted pyrolysis and atomic layer deposition remain limited by process complexity and low scalability. Overcoming these limitations requires innovative strategies in scalable fabrication technologies and catalyst design to fully exploit the unique advantages of dual-metal catalytic systems.

#### 3.1.1. Pt-Based DACs

Platinum-based DACs exhibit outstanding catalytic performance in energy conversion and heterogeneous catalysis, leveraging their tailored atomic configurations and synergistic electronic interactions. These systems achieve high atomic utilization efficiency and demonstrate versatility across multiple applications (e.g., fuel cells and electrolyzers) through optimized metal coordination environments that regulate intermediate adsorption energetics and interfacial charge transfer dynamics.

**Figure 10:**
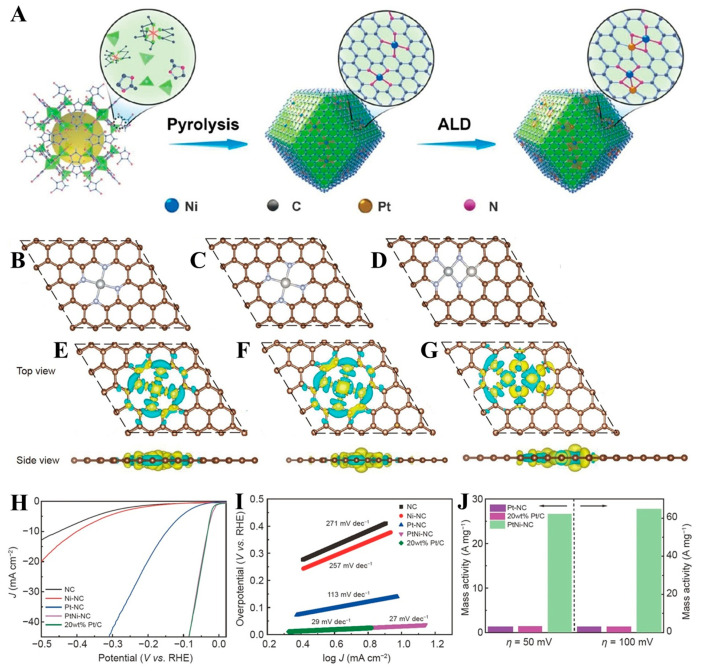
(**A**) A Schematic diagram illustrating the Pt/Ni-NC preparation procedure. The configurations of (**B**) Ni-NC, (**C**) Pt-NC, and (**D**) Pt/Ni-NC after structural optimization are illustrated. The differential charge density diagrams obtained for (**E**) NiN_4_, (**F**) PtN_4_, and (**G**) (Pt/Ni)-N_4_C_2_ are Shown with charge accumulation (cyan) and depletion (yellow). The iso-surface value was 0.004 e A^−3^. (**H**) LSV profiles and (**I**) Tafel profiles, along with (**J**) a comparison with the mass activities of Pt-NC, 20 wt% Pt/C. Reprinted with permission from Ref. [64]. Copyright © 2022, Science China Press and Springer-Verlag GmbH Germany, part of Springer Nature.

Pt/Ni DACs. Pt/Ni DACs overcame the traditional limitations of monometallic/alloy systems via atomic-scale synergy, cost efficiency, and stability enhancement, enabling advanced solutions for clean energy technologies and sustainable chemical processes. Da et al. [64] prepared a platinum/nickel (Pt/Ni) DAC material (Pt/Ni-NC) by atomic layer deposition (ALD) technology. These precursors, such as Zn(NO_3_)_2_, Ni(acac)_2_, and methylimidazole, were dissolved within methanol and homogeneously mixed by magnetic stirring. The resultant mixture underwent solvent evaporation followed by thermal treatment under an argon atmosphere to obtain nitrogen-doped carbon-supported nickel substrates (Ni-NC). The Pt/Ni-NC catalyst exhibited exceptional acidic-condition activity (Figure 10H), demonstrating 21-fold higher mass activity compared to the commercial 20 wt% Pt/C catalyst (Figure 10J). However, its ALD-based fabrication presented challenges: stringent process requirements and high-temperature treatments (≥300 °C) that may compromise atomic-level precision through metal migration, limiting scalable production feasibility.

Liu et al. [65] developed an innovative Ni(OH)_2_-coated platinum catalyst with high-index faceted surfaces, which demonstrated exceptional electrocatalytic performance for HER. The current density (*J*) of the catalyst reached 3.84 mA·cm^−2^, which was 7.5 times greater in comparison to that of the commercial Pt/C catalyst. This was linked to the synergistic catalytic and electron-coupling effects, leading to excellent alkaline HER activity and application potential. Eiler et al. [66] fabricated a mesoporous-structured Pt/Ni alloy nanoparticle catalyst supported on nickel foam through an electrodeposition strategy, employing a platinum-coated titanium mesh counter electrode to establish a homogeneous electric field distribution for achieving uniform nanoparticle deposition. The catalyst exhibited excellent performance in alkaline HER. When the Pt content reached 26 at. %, the catalyst exhibited an eightfold enhancement over bare nickel foam, with low-onset potential, a high Pt utilization rate, and good stability. Pei et al. [67] prepared a Pt_1_/(Co, Ni)(OH)_2_/C single-atom catalyst (SAC) through defect engineering-assisted electrodeposition, where isolated Pt atoms were anchored on defect-rich dual-metal hydroxide nanosheets. The material achieved excellent performance in HER, with an ultra-low *η* value equal to 24 mV vs. RHE, a Tafel slope (TS) equaling 28.7 mV dec^−1^, and remarkable durability. Zhao et al. [68] engineered dual-monodisperse Pt/Ni_3_S_2_ hetero-structured nanocrystals via a hot-injection strategy, featuring precisely controlled Pt nanoparticles (~2 nm) interfacially coupled with Ni_3_S_2_ particles (~9.6 nm). The material demonstrated excellent catalytic performance in both methanol selective oxidation and HER, achieving methanol selective oxidation at 1.45 V vs. RHE (100 mA·cm^−2^, FE > 98%) while simultaneously enabling efficient hydrogen evolution (*η* = 61 mV).

Pt/Fe DACs. Pt/Fe DACs can improve the atom utilization rate and reduce the cost. The electronic interaction between the two atoms can adjust the electronic structure and produce a synergistic effect. Liang et al. [69] developed Pt/Fe DACs through solution-phase synthesis coupled with controlled thermal processing, achieving precise phase engineering guided by the Fe/Pt binary phase diagram. Studies have shown that the introduction of Fe significantly improves the stability and activity of Pt-based catalysts.

Zhou et al. [70] developed nitrogen-bridged Pt/Fe DACs through amino-functionalized carbon nanosheet (CNF-NH_2_) templated synthesis. By precisely coordinating Fe^3+^ and [PtCl_6_]^2−^ precursors at designated amino sites followed by freeze-drying and a controlled annealing process, the team achieved atomic-level Pt/Fe pair configuration with N-mediated electronic coupling. The obtained catalyst exhibited excellent ORR performance. The kinetic current density at the *E*_1/2_ value was about two orders of magnitude greater in comparison to that of commercial Pt/C, and the four-electron path selectivity achieved 99%, showing excellent stability and efficiency in Zn–air batteries.

**Figure 11:**
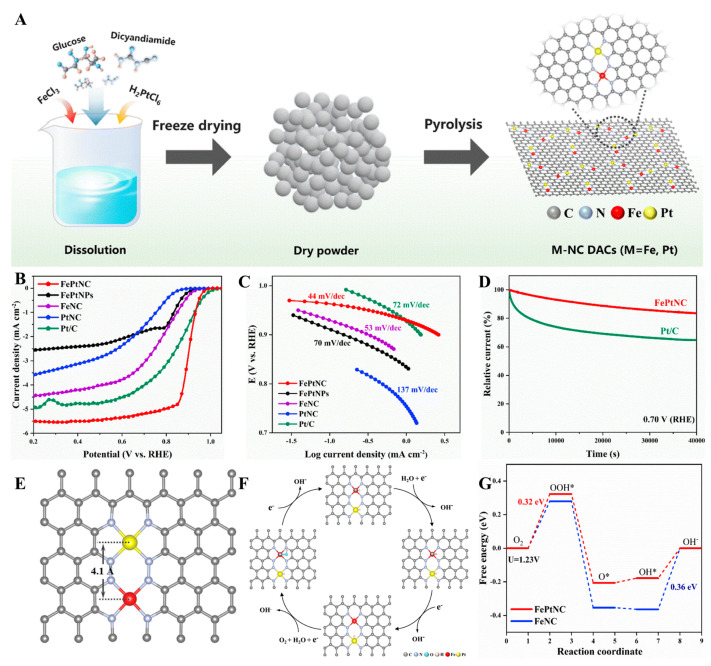
The preparation of Pt/Fe-NC catalysts. (**A**) The preparation of Pt/Fe-NCs is schematically illustrated. The ORR performances of Pt/Fe-NC were assessed in O_2_-saturated 0.1 M KOH. (**B**) A comparison of the ORR polarization plots obtained for Pt/Fe-NC, Pt/Fe-NPs, Fe-NC, Pt-NC, and the commercial Pt/C catalyst. (**C**) Tafel profiles were derived from the polarization plots. (**D**) The *I*–*t* plot of Pt/Fe-NC at 0.70 V vs. RHE was tested alongside Pt/C. The origin of ORR activity within the Pt-Fe dual-site catalyst was discussed. (**E**) The DFT-optimized configuration of Pt/Fe-NC was presented. (**F**) ORR pathways on Pt/Fe-NC were simulated. (**G**) The free-energy diagram of ORR for Pt/Fe-NC was calculated via DFT. * denotes the active site. Reprinted with permission from Ref. [60]. Copyright © The Royal Society of Chemistry 2023.

Atomically dispersed Fe/Pt dual-site catalysts (Fe/Pt-N-Cs) were synthesized by Song et al. [60] through a polymer-assisted pyrolysis strategy, co-embedding Pt and Fe single atoms into N-doped graphene matrices through the thermal decomposition of glucose–dicyandiamide complexes containing FeCl_3_·6H_2_O and H_2_PtCl_6_·xH_2_O precursors (Figure 11). The catalyst not only displayed remarkable stability and ORR activity but also achieved peak power densities equal to 90.33 and 191.83 mW cm^−2^ in Al–air and Zn–air batteries, respectively.

However, challenges existed in the fabrication of Pt/Fe DACs, primarily stemming from high-temperature sintering during thermal processing, which induced Ostwald ripening and active site aggregation. Effective mitigation strategies involve (1) optimizing annealing protocols through lower-temperature/short-duration treatments, (2) employing sinter-resistant supports like SiO_2_@Al_2_O_3_ core–shell structures or N-doped carbon matrices, and (3) implementing spatial confinement techniques via atomic layer deposition. These methods are expected to enhance the stability of the catalyst substantially.

#### 3.1.2. Ir-Based DACs

The Ir/Y DAC system demonstrates significant potential in optimizing the adsorption energetics of reaction intermediates and reducing activation energy barriers through synergistic interactions between the d-orbital electrons of iridium and yttrium coordination.

For OER, the combination of Ir active sites with Y’s electronic modulation capability enables enhanced catalytic activity and operational stability. The incorporation of Y can suppress the migration and agglomeration of Ir atoms, enhance structural stability, and regulate reaction path selectivity through the interfacial charge polarization effect. However, the high cost and resource scarcity of Ir constrain its large-scale application, and the weak metal characteristics of Y may lead to the inactivation of active sites in harsh operating environments, particularly under acidic conditions. Current research predominantly relies on computational simulations, with density functional theory analyses predicting favorable adsorption optimization at Ir/Y interfaces. Experimental validations remain limited, though preliminary studies report a reduction in the value of *η* by approximately 50 mV in alkaline water electrolysis using co-loaded architectures (e.g., Ir/Y @ CNT). Nevertheless, comprehensive durability assessments under prolonged operation are conspicuously absent in the existing literature.

**Figure 12:**
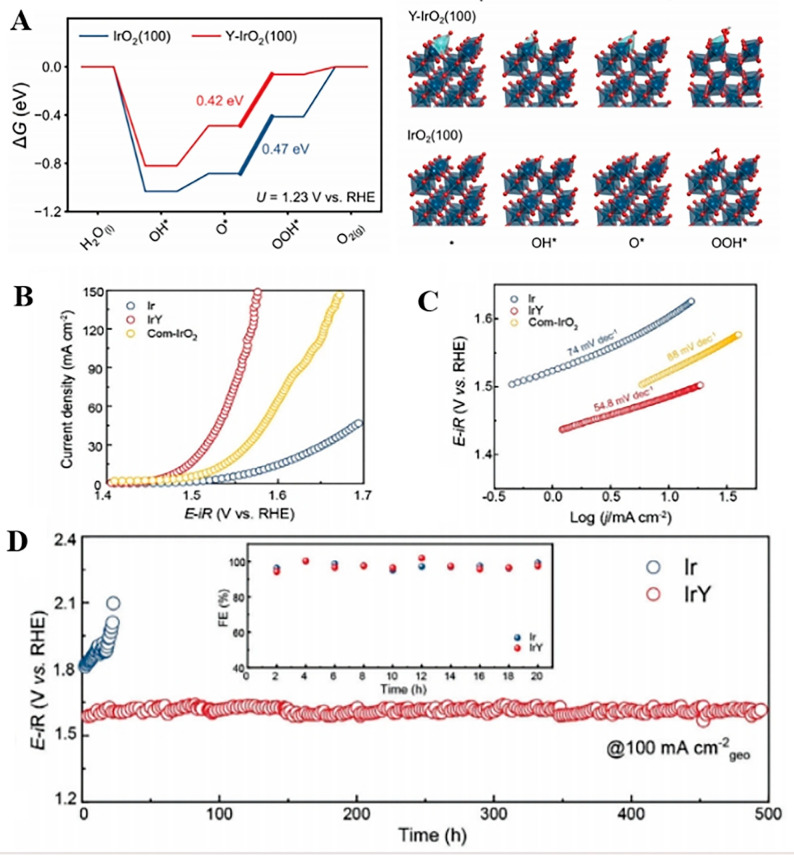
(**A**) Free-energy diagrams of OER on IrO_2_ (100) and Y-doped IrO_2_ (100) were calculated at U = 1.23 V vs. RHE, with bold lines marking the potential-limiting steps (PLSs). Optimized geometries of absorbed OH*, OOH*, and O* on both surfaces, along with their clean counterparts, are illustrated below. * denotes the active site. (**B**) LSV curves compared the OER activities of Ir/Y, Ir, and Com-IrO_2_. (**C**) The TS values for Ir/Y, Ir, and Com-IrO_2_ were derived. (**D**) Chronopotentiometry tests at *J* = 100 mA cm^−2^ within 0.5 M H_2_SO_4_ evaluated the stability of Ir/Y and Ir (inset: Faradaic efficiency monitored via gas chromatography during the initial 20 h). Reprinted with permission from Ref. [72]. Copyright © 2024 Wiley-VCH GmbH.

Zhu et al. [71] prepared Ir/Y DACs using a simple two-step strategy. In this method, Y-MOF material was first formed by the self-assembly of trimesic acid and yttrium chloride hexahydrate under hydrothermal conditions, then iridium chloride hydrate was introduced by in situ adsorption and finally carbonized in the atmosphere of hydrogen and argon to obtain Ir/Y DACs. Ir/Y DACs have excellent stability and activity; their preparation method is simpler in comparison to that of the commercial Pt/C catalyst. Xiong [72] successfully synthesized the Ir/Y alloy using the thermal shock method and rapid Joule heating (1373 K). This method is characterized by a rapid heating and cooling process, which not only ensured a rapid synthesis process but also made the catalyst particles evenly distributed on the substrate, avoiding catalyst agglomeration and forming a uniformly dispersed nanostructure. The Ir/Y alloy catalyst exhibited excellent efficiency within acidic electrolytes, attaining an exceptionally low value of *η* equal to 255 mV at *J* = 10 mA cm^−2^, outperforming conventional IrO_2_ catalysts (290 mV under identical conditions). Its TS value was 54.8 mV dec^−1^ (Figure 12), pointing toward the Heyrovsky step as the RDS. The results revealed that the surface sites of Ir/Y electrocatalysts are dynamically reconstructed into Ir-based oxides during the OER process, wherein metallic surface sites undergo in situ oxidation to form a stabilized but electrochemically active Y-doped IrO_X_ shell.

The Ir/Ni-N-C hetero-diatomic catalyst, successfully synthesized by Liu et al. [73] via an in situ confined pyrolysis strategy, employed a cyanamide–glucose composite as the C/N precursor coupled with sodium iridium chloride and nickel chloride hexahydrate as the metal source. These findings confirm that Ir and Ni atoms were anchored on the carbon support, forming structurally uniform Ir-N_4_ and Ni-N_4_ active centers. The Ir/Ni-N-C catalyst displayed remarkable catalytic activity for ORR within both alkaline and acidic electrolytes. An FeIrN_6_ DAC was developed by Wang et al. [74] through a carbon-support loading method. Synchrotron-based X-ray absorption spectroscopy (EXAFS) verified the absence of a direct Fe-Ir metal bond (Fe-Ir distance > 3A), revealing an isolated N-bridged diatomic architecture. The electrochemical evaluation demonstrated superior acidic OER efficiency, achieving an initial *η* value equal to 1.53 V (vs. RHE) at pH = 1, which is markedly lower than single-atom Ir or Fe counterparts.

Future research needs to focus on non-noble metal substitution (such as the Fe/Y system), in situ characterization techniques (such as the synchrotron radiation XAS analysis of atomic coordination environment), and high-throughput synthesis method innovation to break through the performance–cost balance problem and promote its application in fuel cells and green hydrogen preparation.

#### 3.1.3. Ru-Based DACs

At present, Ru-based DACs have emerged as promising candidates in energy conversion, environmental catalysis, and electrochemical synthesis due to their tunable electronic configurations and dual active site synergy. Through rational design strategies such as the encapsulation-substitution [75,76] method, Ru-based dual-metal systems (e.g., Ru/Co) with tailored metal coordination have been synthesized, demonstrating exceptional catalytic activity and operational stability in electrochemical reactions.

**Figure 13:**
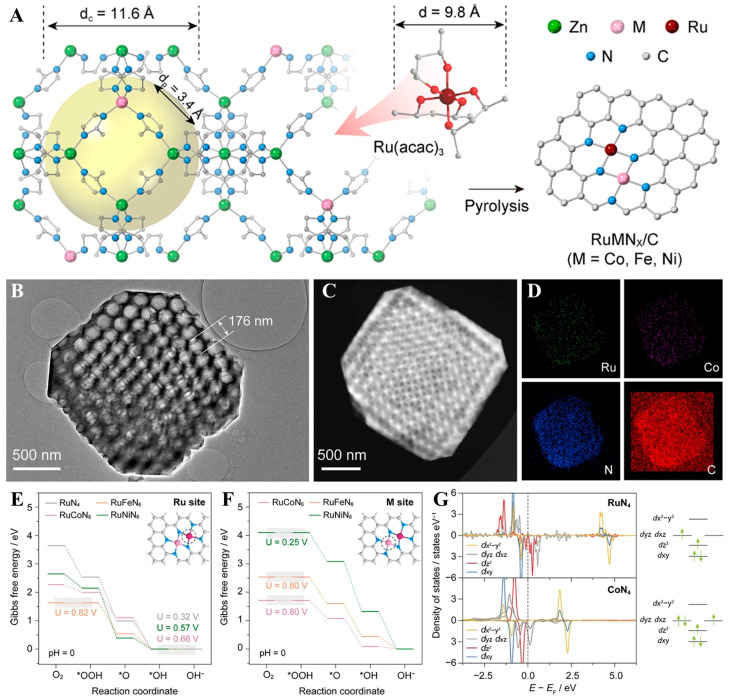
(**A**) The synthetic strategy of Ru/M/N_x_/C was illustrated, where dc, d, and dp represent the diameters of the ZIF-8 cavity, Ru(acac)_3_ molecule, and ZIF-8 aperture, respectively. Structural characterizations included (**B**) low-resolution TEM imaging, (**C**) HAADF-EM imaging, and (**D**) elemental mapping. The reaction free-energy diagrams for ORR on RuMN_6_ (M = Ni, Fe, Co) were calculated at their respective onset potentials (U), showing intermediates adsorbed on either (**E**) Ru sites or (**F**) M sites. (**G**) The projected density of states (PDOSs) of the d orbitals was analyzed for the RuN_4_ configuration. Reprinted with permission from Ref. [76]. Copyright © 2022, American Chemical Society.

Zhang et al. [77] constructed Ru/Co double atomic sites (Ru/Co DAS) on N-doped carbon support via a facile anchoring strategy and combined this with Ru nanoparticles to develop catalysts with multi-component active sites. The theoretical and experimental findings confirmed the atomic-scale behavior of the Ru/Co sites, the strong electron coupling, and the synergistic effect with Ru nanoparticles. This novel electronic structure endowed the catalyst with remarkable stability and HER activity within alkaline environments. Liu et al. [76] fabricated heteronuclear Ru/M (M = Ni, Fe, Co) DACs anchored on nitrogen-doped ordered macroporous carbon substrates via a ZIF-8-templated encapsulation–substitution strategy. The synthesis protocol strategically employed Ru(acac) as a dimensionally constrained Ru precursor, exploiting its intermediate size between ZIF-8′s central cavity (13.6 Å) and pore aperture (3.4 Å) to achieve selective encapsulation within the MOF. At the same time, the selected metal ions (Mn^+^ = Co^2+^, Fe^3+^, and Ni^2+^) were added to replace Zn to obtain M-doped ZIFs. After calcination, M and Ru atoms were embedded within the N-doped carbon matrix (Figure 13). The Ru/Co-N_x_/C catalyst displayed remarkable ORR efficiency for a turnover frequency (TOF) equal to 2.424 s^−1^ and an *E*_1/2_ value equaling 0.895 V (vs. RHE) at 0.7 V, which is indicative of favorable kinetic accessibility and thermodynamic stability. Furthermore, the Ru/Co-N_x_/C-based electrode exhibited superior bifunctional activity in Zn–air batteries, achieving a narrow voltage gap equal to 0.603 V during charge–discharge cycling, outperforming the benchmark Pt/C|RuO_2_ catalyst (0.81 V) and highlighting its potential for high-efficiency energy conversion applications.

By combining density functional theory computations with experiments, Han et al. [78] designed and synthesized a nitrogen-doped porous carbon-supported catalyst (Ru/Ni-NC) with Ru/Ni dual-atomic sites. DFT calculations showed that the dual-atomic site can synergistically regulate the adsorption energy of OH* and H* intermediates to near-optimal values, significantly enhancing the HOR activity. Ru/Ni-NC prepared by the dissolution–carbonization method [79] demonstrated remarkable HOR efficiency within alkaline environments. The material exhibits robust CO tolerance (current retention > 85%) and cycling stability (<6% activity decay following 10,000 cycles), which is associated with the stabilized diatomic coordination structure and electronic synergism between Ru and Ni sites.

**Table 5:** Summarized electrocatalytic activity of noble metal-based DACs.

Catalyst	HER Activity	OER Activity	ORR Activity	Mass Activity	Stability	Ref.
Pt/Ni-NC	*η* = 30 mV TS = 27 mV dec^−1^	NA	NA	MA_1_ = 26.7 A mg^−1^ (*η* = 5.0 mV) MA_2_ = 64.8 A mg^−1^ (*η* = 100 mV)	Att. ≈ 0 (2000 cycles of CV test)	[64]
Fe_x_/Pt_y_-CN	Fe_0.1_/Pt_0.9_-CN *η* = 29 mV TS = 30 mV dec^−1^ Fe_0.25_/Pt_0.75_-CN *η* = 36 mV TS = 40 mV dec^−1^	NA	NA	MA = 3.8 A mg^−1^ Pt (Fe_0.1_/Pt_0.9_-CN, *η* = 50 mV)	*η* increased by 51 mV (at 10 mA cm^−2^)	[69]
Pt/Ir	NA	*J* = 26.8 μA·cm^−2^ (0.5 M H_2_SO_4_, *η* = 0.4 V)	NA	M_ALD_ = 557 A·g^−1^ (*η* = 0.39 V)	*J* decreased by 8% (stored in 1.6 M vanadium electrolyte for 1 day)	[80]
Pt_3.6_/Ni-S NWs	TS = 114.7 mVdec^−1^	NA	NA	MA = 4.37 mA/mg Pt (*η* = 70 mV)	NA	[81]
Ce/Se-NC	TS = 51.28 mV·dec^−1^	NA	*E*_1/2_= 0.886 V vs. RHE	NA	*E*_1/2_ decreased 11 mV (8000 cycles) Att. ≈ 0 (at 10 mA·cm^−2^)	[82]
Pt/Co-C	NA	NA	TS = 93–113 mVdec^−1^ I_0(0.5at%Co)_ = 1.13 × 10^−5^ A/cm^2^ I_0(1at%Co)_ = 1.59 × 10^−5^ A/cm^2^	MA_(0.5at%Co)_ = 104 mA/mg MA_(1at%Co)_ = 124 mA/mg	Att. ≈ 0 (500 h of stability test)	[83]

Here, NA denotes non-availability; TS denotes the Tafel slope; I_0_ denotes the exchange current density; *J* denotes the current density; Att. denotes attenuation; and *η* denotes overpotential.

### 3.2. Non-Noble Metal DACs

In recent years, non-noble metal DACs have gained immense popularity owing to their low cost, high atomic utilization, and dual-metal synergistic effect. As shown in Table 6, these catalysts enable the precise modulation of electronic configurations and coordination environments through the atomic-level engineering of dual transition metal active sites, thereby substantially enhancing catalytic selectivity and activity in critical reactions such as O_2_ reduction, H_2_ evolution, and organic transformations. However, there are still some challenges in the synthesis process, such as the controlled dispersion of dual-metal atoms, structural stability, and the precise regulation of the active site structure. At the same time, the in-depth understanding of the reaction mechanism is still insufficient, which hinders its transformation to practical application. In general, future research needs to further optimize the preparation methods, strengthen theoretical calculations, and in situ experimental analysis to obtain accurate structural design and long-term stable and efficient catalytic performance breakthroughs.

#### 3.2.1. Fe-Based DACs

As an important branch of non-noble metal catalytic systems, Fe-based dual-metal atom catalysts (Fe-DACs) have shown huge potential in the field of energy catalysis and environmental pollution control. Through the electronic synergistic effect and geometric configuration regulation of dual-metal sites (such as Fe/Co, Fe/Ni), Fe-DACs can optimize the adsorption free-energy distribution of intermediates and significantly enhance catalytic activity. Furthermore, Fe-DACs regulate the spin state of active sites through axial-OH ligands within acidic environments, restricting the dissolution of Fe atoms and making the stability of PEMFC exceed 1000 h@1 A/cm^2^, which is nearly three times greater compared to that of traditional Fe-N-C SACs [84].

**Figure 14:**
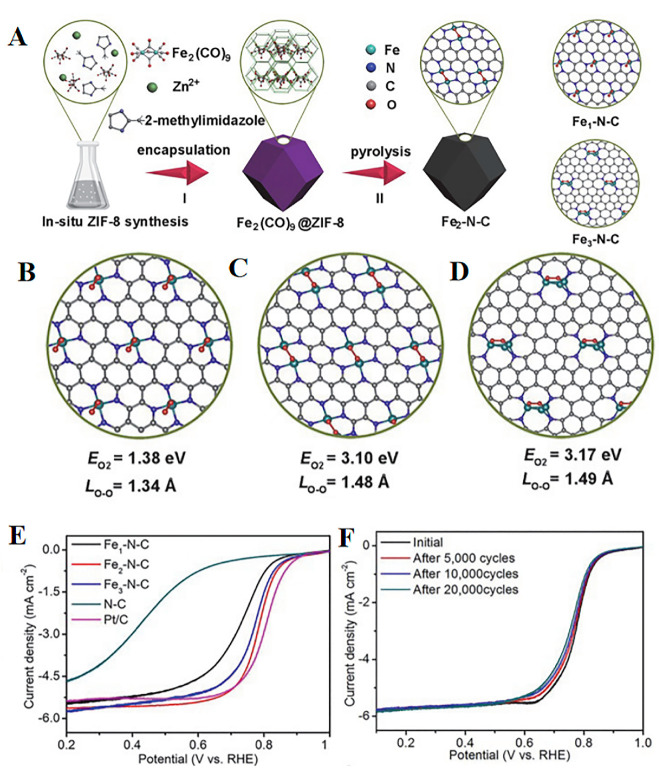
(**A**) The two-step preparation of Fe_2_-N-C. The adsorption energies (E_O2_), optimized adsorption configurations, and O-O bond lengths (LO-O) for O_2_ on Fe_1_-N-C (**B**), Fe_2_-N-C (**C**), and Fe_3_-N-C (**D**). (**E**) ORR polarization plots in O_2_-saturated 0.5 M H_2_SO_4_. (**F**) The ORR durability test results for Fe_2_-N-C following 5000, 10,000, and 20,000 cycles at 50 mVs^−1^ (0.6–1.0 V). Reprinted with permission from Ref. [85]. Copyright © 2023 Dalian Institute of Chemical Physics, the Chinese Academy of Sciences. Published by Elsevier B.V. All rights reserved.

Ye et al. [85] synthesized Fe_2_-N-C DACs via precursor pre-selection and the ZIF-8 encapsulation strategy. They used Fe_2_(CO)_9_ as a binuclear precursor, encapsulated it in the pores of ZIF-8, and obtained the Fe_2_-N-C catalyst following high-temperature pyrolysis. The Fe_2_-N_6_ site absorbs O_2_ molecules through a bridge, significantly weakening the O-O bond (the bond length stretched from 1.23 to 1.40 Å), and promoting the four-electron ORR pathway. As shown in Figure 14. E-F, its *E*_1/2_ value within an acidic environment reached 0.78 V (vs. RHE), and the stability test only attenuated 20 mV following 20,000 cycles. 

**Figure 15:**
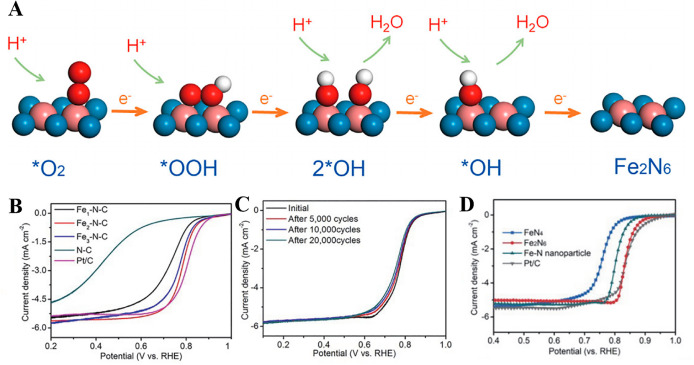
(**A**) Proposed ORR routes on the planar Fe_2_N_6_ structure. (**B**) ORR polarization plots in O_2_-saturated 0.5 M H_2_SO_4_. (**C**) ORR durability evaluation of Fe_2_-N-C following 5000–20,000 cycles at a scan rate (*v*) equal to 50 mV s^−1^ (0.6–1.0 V). (**D**) The ORR polarization plots obtained for the isolated FeN_4_, planar Fe_2_N_6_, and Fe-N nanoparticles within 0.5 M H_2_SO_4_. * denotes the active site. Reprinted with permission from Ref. [86]. Copyright © 2023 Dalian Institute of Chemical Physics, the Chinese Academy of Sciences. Published by Elsevier B.V. All rights reserved.

A configuration of planar Fe_2_N_6_ catalytic sites was reported by Zhang et al. [86], which was achieved through the thermally induced atomic migration of isolated FeN_4_ moieties on a graphitized carbon substrate. This structural evolution dynamically coupled the original FeN_4_ single-atom sites with the newly formed Fe_2_N_6_ diatomic configurations, driven by high-temperature-driven migration processes that enabled the precise spatial reorganization of metal coordination centers. In situ EXAFS showed that the Fe-Fe bond length shrank from 2.37 Å to 2.25 Å during the ORR process, and the OOH* intermediate was bridged to accelerate the O-O bond cleavage. As illustrated in Figure 15, the catalyst demonstrated an *E*_1/2_ value equal to 0.84 V (vs. RHE) in 0.5 M H_2_SO_4_ and delivered a peak power density equal to 845 mW cm^−2^ in fuel cell testing. The surface-exposed active sites on the carbon matrix significantly enhanced mass transport efficiency. However, the thermal activation process during synthesis induced partial Fe atom agglomeration into nanoparticles, which highlighted the need for the precise optimization of synthesis parameters to balance atomic dispersion with structural stability.

An Fe/Zn-N-C DAC was developed by Li et al. [87] via a hydrothermal–pyrolysis approach, employing CoFe_2_O_4_ as a structural template to adsorb pyrrole monomers, followed by controlled pyrolysis to establish Zn-N_4_ and Fe-N_4_ dual active sites. Zn incorporation effectively downshifted the d-band center of Fe and lowered the OH* adsorption energy. The catalyst demonstrated an acidic ORR *E*_1/2_ value equal to 0.808 V (vs. RHE) with enhanced durability, exhibiting only 28 mV of decay after 5000 accelerated durability test cycles. Nevertheless, the inherently low catalytic activity of Zn species likely compromised the overall atomic utilization efficiency.

The precise synthesis of Fe-based DACs still faces challenges in achieving atomic dispersion, requiring complex supports (e.g., N-doped carbon nanotubes and MOFs) and harsh preparation conditions that compromise batch stability. The dynamic reconstruction–reactivity correlation remains unclear, necessitating the in situ synchrotron XAS analysis. Future efforts should prioritize high-throughput synthesis, mechanistic studies of site evolution, and non-noble metal systems to overcome cost–performance trade-offs for applications in fuel cells and green hydrogen production.

Fe/Co DACs. Fe/Co DACs [88,89,90] make full use of the dual-metal synergistic effect and high atomic utilization rate and optimize the catalytic active sites by finely regulating the electronic interaction between Fe and Co in order to show significant activity and high selectivity in electrocatalytic and photocatalytic reactions. However, this type of catalyst often faces the challenge of precise dispersion and stability control of diatomic sites during the synthesis process and is prone to agglomeration or structural reconstruction, which limits the durability of long-term use. In addition, the complexity of the catalytic mechanism also increases the difficulty of the in-depth analysis of the structure–activity relationship. At present, researchers are trying to explore its structure–activity mechanism through new in situ characterization tools, synthesis methods, and theoretical calculations, thereby accelerating their implementation in energy conversion systems and sustainable chemical synthesis.

**Figure 16:**
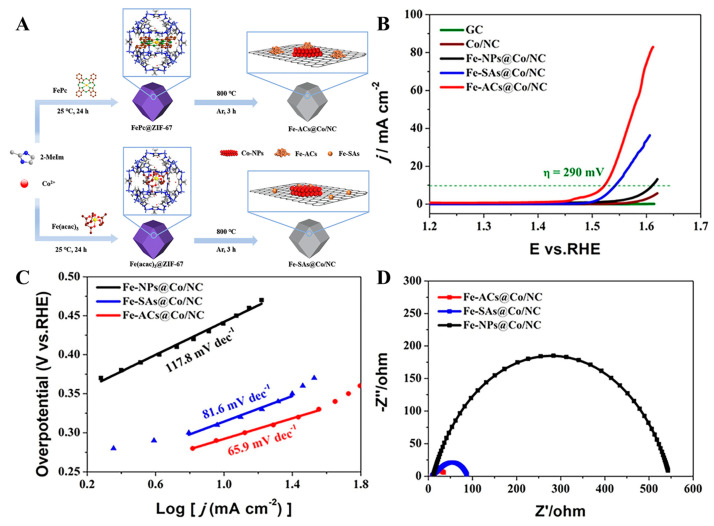
(**A**) Synthesis of Fe-Acs @ Co/NC and Fe-SAs @ Co/NC. (**B**) OER polarization curves (iR-corrected) of GC, Fe-NPs @ Co/NC, Co/NC, Fe-Acs @ Co/NC, and Fe-SAs @ Co/NC within 1 M KOH at *v* = 5 mV s^−1^. (**C**) TS values and (**D**) EIS Nyquist plots at *η* = 350 mV for Fe-NPs @ Co/NC, Fe-Acs @ Co/NC, Fe-SAs @ Co/NC, and Fe-Acs @ Co/NC. Reprinted with permission from Ref. [91]. Copyright © 2023 The Authors.

The fundamental challenge in fabricating Fe/Co DACs lies in achieving atomic-level dispersion with a durable coordination stabilization of Fe/Co diatomic pairs, which dictates their synergistic catalytic functionality. Du et al. [91] engineered Fe-Acs @ Co/NC composite catalysts through the molecular encapsulation of iron acetylacetonate (Fe(acac)_3_) and iron phthalocyanine (FePc) within ZIF-67 nanocages, followed by pyrolysis at 800 °C. Electrochemical characterization revealed superior OER performance for Fe-Acs @ Co/NC in 1 M KOH, achieving a low *η* value equaling 290 mV at 10 mA cm^−2^, which outperformed Fe-NPs @ Co/NC (380 mV) and Fe-Sas @ Co/NC (310 mV). The optimal kinetics were evidenced by its minimal TS value equal to 65.9 mV dec^−1^ (Figure 16). DFT calculations identified Co nanoparticles as active centers, where Fe clusters synergistically modulate oxygen intermediate adsorption-free energy through electronic interactions, thereby enhancing OER efficiency.

Dongyoon Woo et al. [92] developed Fe/Co-LMC catalysts through directed self-assembly, achieving the atomic-level anchoring of Fe/Co dual sites on anisotropic mesoporous carbon matrices. This architecture demonstrated exceptional bifunctional oxygen redox activity in natural seawater electrolytes. Bai’s [93] group established an electrochemical activation protocol that reconstructs Co-N-C single-atom precursors in Fe-containing alkaline electrolytes, generating Fe/Co-N-C catalysts with atomically defined dual-metal sites. The optimized catalyst attained a record turnover frequency (TOF) equal to 4.2 s^−1^ among non-precious OER catalysts. Combined experimental and theoretical analyses revealed Fe-induced coordination reconstruction around Co centers, which synergistically optimized oxygen intermediate adsorption through electronic structure modulation, elucidating the dual-atom cooperative mechanism underlying enhanced reaction kinetics.

Fe/Ni DACs. Fe/Ni DACs have been an important extension in the field of SACs over the last few years. The rational integration of Fe/Ni dual-metal active centers through precise coordination engineering enables synergistic electronic modulation, simultaneously optimizing catalytic performance metrics, including activity, selectivity, and structural durability. It shows great potential in the fields of energy conversion and environmental catalysis. The development of effective and stable electrocatalysts for acidic overall water splitting is very important for sustainable hydrogen production, but it still faces enormous challenges.

Cheng [94] prepared an atomically dispersed dual-metal Fe/Ni catalyst anchored on N-doped carbon nanotubes (N-CNTs) via an improved one-pot synthesis strategy. Catalysts with various Fe:Ni weight ratios (from 1:0 to 0:1) were synthesized by appropriately modifying the ratio of Ni and Fe precursors. The Fe/Ni catalyst (Fe:Ni ≈ 2:1~1:1) exhibited optimal ORR performance in alkaline media, outperforming monometallic catalysts. Wu [95] prepared a series of Fe-based dual-metal–organic framework (MOF) materials (Fe_2_M-MIL-88B, M was Ni, Co, Mn) using a solvothermal synthesis strategy. By precisely regulating the metal nodes of MOFs, dual-metal active sites such as Fe_2_Ni, Fe_2_C, and Fe_2_Mn were successfully constructed. The prepared Fe_2_M-MIL-88B catalyst delivered the best efficiency in OER. The XPS results and DFT computations revealed that there was a strong electronic coupling effect present between Fe and Ni, and some electrons were transported from Ni^2+^ to Fe^3+^ through bridging oxygen O^2−^, which significantly optimized the electronic structure of the metal active sites, thus enhancing the intrinsic catalytic activity of OER.

**Figure 17:**
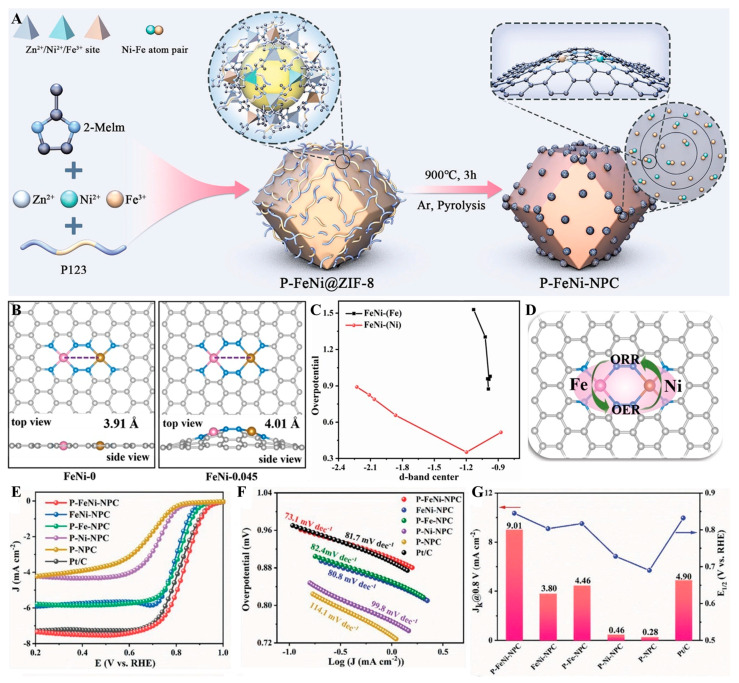
(**A**) A synthesis schematic of P-Fe/Ni-NPC. (**B**) Structural models of FeNi-0 and FeNi-0.045. (**C**) Plots of *η* against the d-band center with curvatures for Fe/Ni-(Fe) and Fe/Ni-(Ni). (**D**) The proposed Fe-Ni metal pair configuration in P-Fe/Ni-NPC. (**E**) The polarization plots of various catalysts. (**F**) TS values and (**G**) performance comparison (*E*_1/2_ and *J*_k_ @ 0.80 V) between P-Fe/Ni-NPC and reference catalysts. Reprinted with permission from Ref. [96]. Copyright © 2024 Wiley-VCH GmbH.

A Fe/Ni metal atom pair catalyst (P-Fe/Ni-NPC) was developed by Qin et al. [96] and was protected by a hierarchical porous cyclic carbon network through a mediator-assisted metal–organic framework derivatization strategy. As shown in Figure 17, Fe^3+^ and Ni^2+^ were co-incorporated into ZIF-8 precursors with Pluronic P123, directing pyrolysis-induced concentric carbon frameworks that atomically anchored Fe/Ni pairs via N-coordination. This structure ensured the uniform distribution and efficient utilization of Fe-N_4_ and Ni-N_4_ active sites. The P-Fe/Ni-NPC catalyst demonstrated exceptional bifunctional oxygen electrocatalytic activity, achieving an OER *η* value equal to 320 mV and an ORR *E*_1/2_ value equaling 0.845 V vs. RHE and at 10 mA cm^−2^ within alkaline environments. These values approach commercial benchmarks (Pt/C: 0.840 V; RuO_2_: 270 mV) while exhibiting a remarkably low ΔE (0.705 V), surpassing most non-precious bifunctional catalysts reported.

The electronic structure of Fe/Ni DACs was optimized via the synergistic impact between Ni and Fe atoms, which improved the efficiency of oxygen adsorption and desorption. Although the prepared materials have excellent properties, the heat treatment method requires precise control of temperature and atmosphere, and the preparation conditions are harsh, so large-scale production may be difficult.

**Figure 18:**
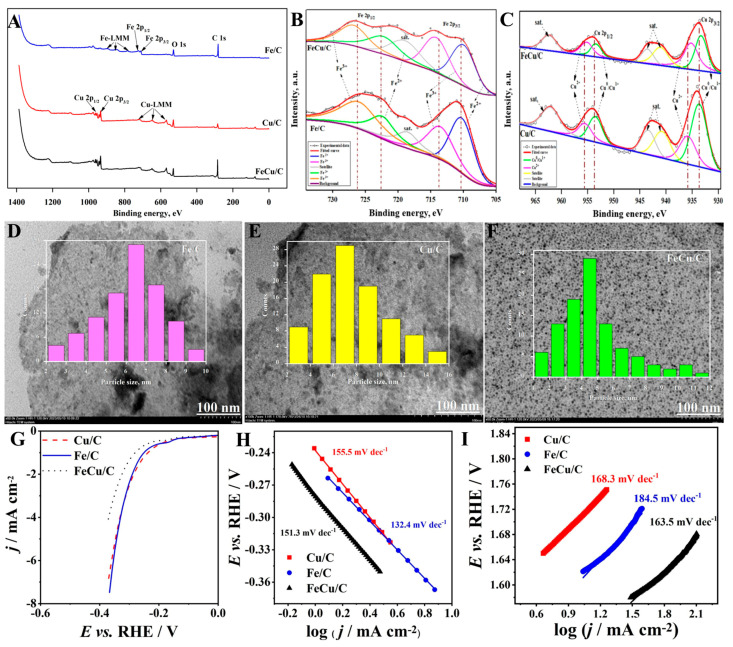
(**A**) The XPS spectra of synthesized catalysts. (**B**) Fe 2p and (**C**) Cu 2p XPS deconvolutions of Fe/C and Fe/Cu-C. TEM images and particle size distributions of (**D**) Fe/C, (**E**) Cu/C, and (**F**) Fe/Cu-C. HER performance: (**G**) polarization plots, (**H**) TS values, and (**I**) Nyquist plots (1.8 V) with equivalent circuit insets for Fe/Cu-C, Cu/C, and Fe/C. Reprinted with permission from Ref. [97]. Copyright © 2024 The Author(s). Published by Elsevier B.V.

Fe/Cu DACs. Fe/Cu DACs have emerged as potential contenders for energy conversion technologies, particularly in electrochemical water splitting and Zn–air battery systems. Jelena Georgijević’s [97] team developed Fe/Cu-N-C catalysts via ionic liquid-assisted carbonization, demonstrating exceptional bifunctional activity for alkaline water electrolysis (Figure 18). The catalyst attained a low OER *η* value equal to 280 mV at 50 mA cm^−2^ with 95% current retention after 24 h of chronoamperometric testing. Electrochemical impedance spectroscopy revealed ultra-low charge-transfer resistance (<5 Ω·cm^2^), while in situ XANES analysis suggested that Fe site dissolution–redeposition dynamics are a stability preservation mechanism through continuous active site regeneration.

**Figure 19:**
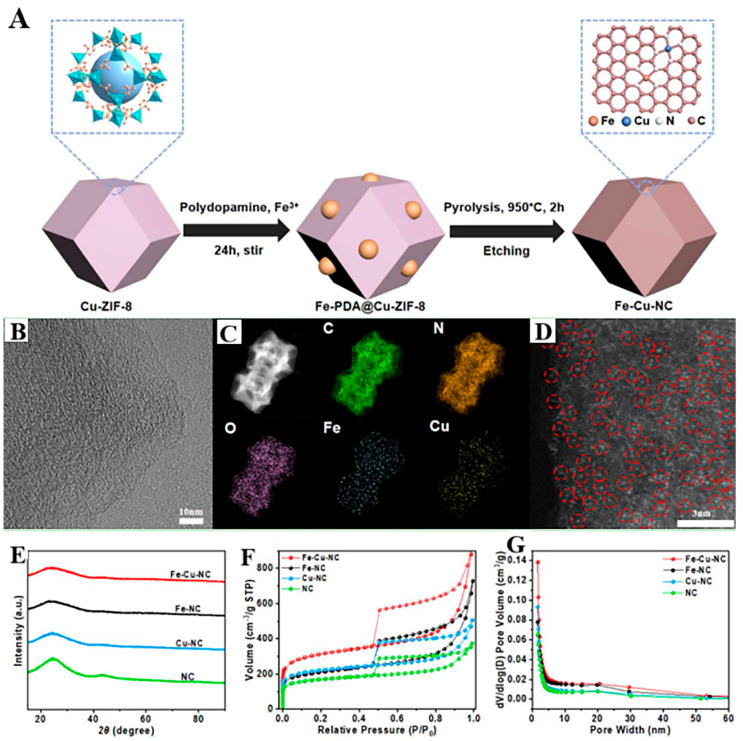
(**A**) A schematic illustration of the Fe/Cu-NC synthesis process. (**B**–**D**) The HR-TEM image, elemental mapping, and AC-HAADF-STEM image obtained for Fe/Cu-NC. (**E**) The XRD profiles obtained. (**F**) The N_2_ desorption/adsorption isotherms and (**G**) pore size distributions obtained for Fe/Cu-NC, Fe-NC, Cu-NC, and NC. Reprinted with permission from Ref. [98]. Copyright © 2025 Copyright Clearance Center, Inc. All rights reserved.

An Fe/Cu-NC dual SAC was developed by Wang et al. [98] via sequential coordination engineering on ZIF-8 templates, co-embedding Cu(acac)_2_, and Fe–polydopamine complexes followed by pyrolysis and acid etching. This hierarchical micro/meso-macroporous architecture (Figure 19) exhibited a 40% enhanced BET surface area (1280 m^2^ g^−1^) versus monometallic analogs, enabling ultra-high Fe site density (1.2 × 10^20^ sites g^−1^). Electrochemical characterization revealed superior ORR efficiency with an *E*_1/2_ value equal to 0.890 V vs. RHE (35 mV surpassing Pt/C) and a reduced TS value equal to 68 mV dec^−1^. DFT calculations verified Fe/Cu dual sites that synergistically optimized *OOH adsorption through d-band center modulation. When deployed in zinc–air batteries, the catalyst delivered a 183.1 mW cm^−2^ peak power density, a 787 mAh g^−1^ specific capacity, and >200 h cycling stability (<3% decay), outperforming commercial Pt/C + RuO_2_ counterparts.

A dual-metal SAC was designed by (Fe/Cu-NC) Gong et al. [99] with atomically dispersed Cu_2_N_6_ and FeN_4_ sites by hydrothermal-ball-milling-assisted secondary calcination. The Fe/Cu-NC catalyst exhibited a hierarchical porous architecture comprising spherical assemblies with abundant micro/mesopores (BET surface area: 2883.7 m^2^ g^−1^), facilitating active site accessibility and electrolyte transport. Synergistic electronic interactions between Cu_2_N_6_ and FeN_4_ configurations induced collective d-band center upshifting, as evidenced by operando XANES analysis. This electronic modulation endowed the catalyst with superior O_2_ reduction activity within alkaline and acidic environments.

#### 3.2.2. Co-Based DACs

Beyond Fe-based systems, Co-DACs have received a lot of attention owing to their superior catalytic performance and tunable electronic configurations. The strategic pairing of Co with secondary metals (e.g., Mn, Ni) enabled precise charge redistribution through d-band center modulation, which optimized intermediate desorption/adsorption energetics and reduced activation barriers in redox processes, particularly enhancing OER efficiency and selective pollutant degradation.

**Figure 20:**
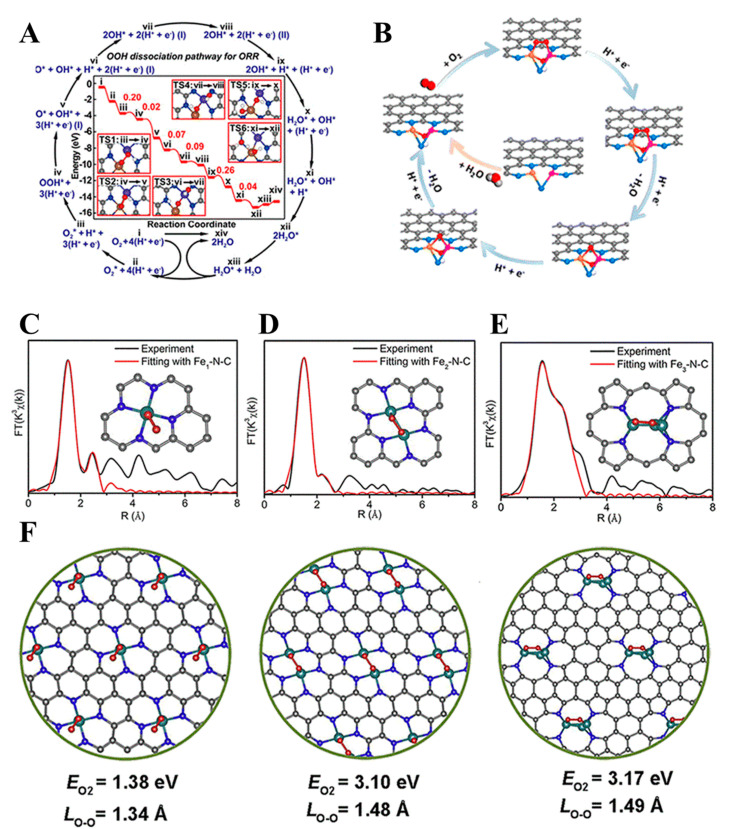
(**A**) DFT-calculated ORR intermediate/transition state energies on (Fe, Co)/N-C. (**B**) The proposed ORR mechanism at Fe/Co-N_5_-OH. (**C**–**E**) The k_3_-weighted Fourier transform (black: experimental Fe K-edge EXAFS spectrum; red: fitted curve) for Fe_1_/Fe_2_/Fe_3_-N-C with optimized structural models (insets; Fe: green, C: gray, N: blue, O: red). (**F**) Superoxo-like adsorption at Fe_1_-N-C, peroxo-like adsorption at Fe_2_-N-C, and peroxo-like adsorption at Fe_3_-N-C. Reprinted with permission from Ref. [101]. Copyright © The Royal Society of Chemistry 2022.

A self-supporting amorphous nanoporous Ni-Co phosphide catalyst was prepared by Yao et al. [100] via an electrochemical dealloying strategy. The material demonstrated remarkable HER efficiency within a 1 M KOH electrolyte. The optimized catalyst achieved a low *η* value equal to 114 mV at *J* = 10 mA cm^−2^ with a TS value equaling 57.3 mV dec^−1^ while maintaining 95% initial activity after a 50 h stability test. Studies showed that the synergistic impact of Ni/Co dual-metal phosphides significantly enhanced the inherent activity of the active site, with a mass activity that was three-fold higher compared to monometallic counterparts. An Fe/Co dual SAC (Fe/Co-NC) was developed by Wang et al. [101] via the FeCl_3_-mediated pyrolysis of Co/Zn-MOF precursors. Aberration-corrected HAADF-STEM and XAFS analyses confirmed atomic Fe/Co pairs with FeN_3_-CoN_2_ coordination configurations embedded in N-doped carbon matrices (Figure 20). This dual-site synergy endowed the catalyst with superior acidic ORR performance, exhibiting an onset potential equal to 1.06 V and an *E*_1/2_ value equal to 0.863 V vs. RHE, surpassing commercial Pt/C by 28 mV.

**Figure 21:**
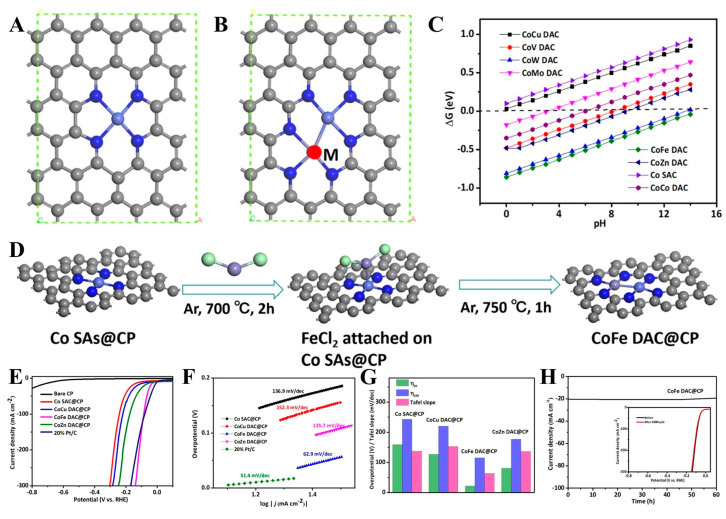
A structural diagram of Co SAC (**A**) and Co/M DACs (**B**), in which dark blue represents N, gray represents C, white represents H, and light blue represents Co; M = V, W, Cu, Mo, Zn, Co, and Fe. (**C**) Gibbs free energy for the H adsorption (∆GH*) on single atoms of the Co catalyst and various Co/M DACs. A schematic diagram illustrating the generation of Co/Fe DAC (**D**); HER activity evaluation in 1.0 M KOH (**E**–**H**): polarization plots (**E**) and TS values (**F**); catalytic activity comparisons (**G**); and a stability test after 5000 cycles (−0.95 to −1.10 V vs. Ag/AgCl) (**H**). Reprinted with permission from Ref. [103]. Copyright © 2022 Elsevier B.V. All rights reserved.

A Co/Mn DAC (CoN_2_S/MnN_3_-N-C) was fabricated by Zhao et al. [102] through an adsorption–pyrolysis strategy, where asymmetric charge polarization between Co and Mn sites was achieved through sulfur-modulated coordination environments. This catalyst demonstrated superior O_2_ reduction activity with an *E*_1/2_ value equal to 0.901 V vs. RHE, exceeding the commercial Pt/C catalyst. Jiao et al. [103] developed Co/Cu and Co/Fe DACs through DFT-guided chemical vapor deposition. These DACs exhibited adjacent Co/M (M = Cu; Fe) sites with optimized hydrogen adsorption energies, enabling remarkable HER efficiency in both alkaline and acidic environments (Figure 21).

Although Co/Cu and Co/Fe DACs demonstrated promising activity, their mass activity remained inferior to commercial 20% Pt/C due to limited active site density. To address these limitations, three strategic methods emerged: (1) developing scalable synthesis routes (e.g., pulsed laser ablation) to achieve cost-effective mass production, (2) the atomic-level engineering of coordination microenvironments to enhance durability, and (3) integrating interfacial engineering strategies with compatible electrolytes to optimize reaction pathways through multiphase synergy.

**Table 6:** Summarized electrocatalytic activity of non-noble metal DACs.

Catalyst	HER Activity	OER Activity	ORR Activity	Mass Activity	Stability	Ref
Fe_2_-N-C	TS = 67 mV dec^−1^ E_onset_ = 0.0 V vs. RHE (0.1 mol L^−1^ HClO_4_)	*E*_1/2_ = 0.37 Vvs. RHE (0.1 mol L^−1^ KOH)	*E*_1/2_ = 0.78 V vs. RHE (0.5 mol/L H_2_SO_4_ )	MA = 0.45 A mg^−1^ (0.5 mol L^−1^ H_2_SO_4_)	*E*_1/2_ decreased 20 mV (20,000 cycles)	[85]
Fe_2_N_6_	TS = 67 mV dec^−1^ E_onset_ = 0.0 V vs. RHE (0.1 mol L^−1^ HClO_4_)	E_onset =_ 1.5 V vs. RHE (0.1 mol L^−1^ KOH)	*E*_1/2_ = 0.84 V vs. RHE (0.5 mol/L H_2_SO_4_)	MA = 0.45 A mg^−1^ (0.5 mol L^−1^H_2_SO_4_)	*E*_1/2_ decreased 24 mV (10,000 cycles)	[86]
Fe/Zn-N-C	TS = 75 mV dec^−1^ (0.1 mol L^−1^ HClO_4_) E_onset_ = 0.0 V vs. RHE (0.1 mol L^−1^ HClO_4_)	*E*_1/2_ = 0.37 V vs. RHE (0.1 mol L^−1^ KOH)	*E*_1/2_ = 0.808 V vs. RHE (0.1 mol/L HClO_4_)	MA = 0.30 A mg^−1^ (0.1 mol L^−1^ HClO_4_)	*E*_1/2_ decreased 25 mV (2000 cycles)	[87]
Fe/Co-N-C	TS = 66 mV dec^−1^ (0.1 M HClO_4_)	E_onset_ = 1.06 V vs. RHE (0.1 M HClO_4_)	*E*_1/2_ = 0.863 V vs. RHE (0.1 M HClO_4_)	MA = 0.35 A mg^−1^ (0.1 M HClO_4_)	*E*_1/2_ decreased 20 mV (50,000 CV cycles test)	[93]
Fe/Cu-C	TS = 152 mV dec^−1^ E_onset_ = −0.335 V ƞ = 150 mV (At 10 mA cm^−2^)	TS = 163.5 mV dec^−1^ E_onset_ = 1.319 V ƞ = 325 mV (at *J* = 10 mA cm^−2^) *J* = 83.8 mA cm^−2^ (At *ƞ* = 400 mV)	NA	NA	Δ*J*_(_*_CUD_*_)_ = 0 (at a potential of −0.35 V, 24 h chronoamperometry current test)	[97]
Fe-/Nb-C-SNC	NA	* η * = 390 mV (at *J* = 10 mA cm^−2^)	* E *_ 1/2 _ = 0.922 V (vs. RHE) *J* = 15.6 mA cm^−2^ at 0.9 V	TOF = 4.64 e^−^ site^−1^ s^−1^ PPD_(ZAB)_ = 314 mW cm^−2^ PPD_(HEMFC)_ = 1.18 W cm^−2^	Δ*J*_(_*_ACD_*_)_ = 0 (at a potential of 1.8 V, 24 h chronoamperometry current test) *E*_1/2_ decreased 20 mV (50,000 CV cycles test)	[104]

Here, E_onset_ denotes initial potential; TS denotes the Tafel slope; NA denotes non-availability; TOF denotes turnover frequency; ACD denotes anode current density; CUD denotes cathode current density; and PPD denotes peak power density.

## 4. MACs

Noble metal multi-atom catalysts (such as Ir/Ru/Co, Pt/Pd/Rh, and other three-atom and above systems) show significant advantages in the field of catalysis owing to the synergistic impact of multi-metals and the high tunability of their electronic structure. The electronic coupling and geometric configuration synergy between multiple atoms can accurately optimize the adsorption energy of intermediates. For example, multi-component synergy in high-entropy alloys (such as Fe/Co/Ni/Ir/Ru) significantly improves the activity of OER and reduces the *η* value. At the same time, the multi-atomic dispersion structure can inhibit the agglomeration of noble metals and enhance stability [105]. In addition, the inherent high conductivity and corrosion resistance of noble metals are retained in multi-atomic systems so that they can maintain catalytic activity under harsh conditions.

### 4.1. Noble Metal MACs

**Figure 22:**
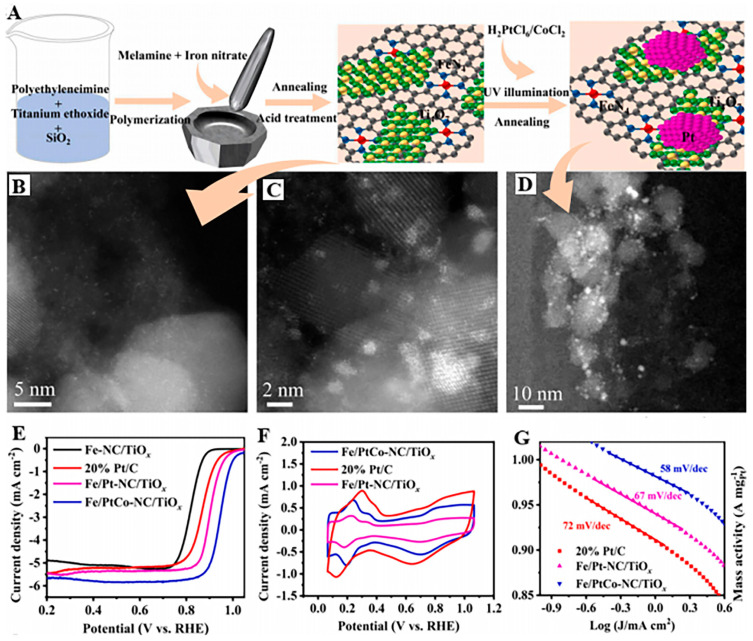
The structural and electrochemical analyses of catalysts: (**A**) Fe/Pt-NC/TiOx synthesis process. (**B**,**C**) The TEM images obtained for Fe/Pt-NC/TiOx and Fe-NC/TiOx (pre-annealing). (**D**) The obtained HAADF-STEM image. Electrochemical performance in 0.1 M KOH: (**E**) The obtained ORR polarization plots (1600 rpm). (**F**) CVs in N_2_-saturated electrolytes (Fe/Pt-NC/TiOx, Fe/Pt/Co-NC/TiOx, and 20% Pt/C). (**G**) Tafel plots comparing catalyst activities. Reprinted with permission from Ref. [107]. Copyright © 2022 Elsevier B.V. All rights reserved.

Noble metal MACs (e.g., Pt-, Pd-, and Ru-based systems) leverage unique electronic configurations to significantly reduce reaction activation energy barriers and enhance intrinsic reaction kinetics. Alloying strategies with low-cost metals (Cu and Co) enable both noble metal load reduction and electronic structure optimization via strain/ligand effects (Table 7). In the electrocatalytic oxidation of methanol, Pt-based catalysts can substantially enhance the reaction rate and promote the effective conversion of methanol. Deng’s [106] team prepared Pt/Ru/Ni-NWs-C ternary alloy nanowire catalysts using the direct grinding and co-reduction strategy. The material had a 1D nanowire structure with a high aspect ratio and good dispersion, and its regular morphology effectively exposed abundant active sites. It exhibited excellent performance in the methanol oxidation reaction (MOR). The ternary components with density gradient distribution inhibit CO poisoning through electronic effects and synergistically improved catalytic stability. However, in the synthesis strategy of such materials, the co-reduction method requires precise control of experimental conditions, such as heat treatment temperature and time, which poses a challenge to large-scale production. Luo et al. [107] prepared Fe/Pt/Co-NC/TiO_X_ materials using the co-precipitation method and wet impregnation method combined with light and calcination strategy. The optimized Fe/Pt/Co-NC/TiO_x_ catalyst demonstrated exceptional performance in ORR (Figure 22), with an *E*_1/2_ value equal to 0.948 V, a mass activity equaling 3.69 A mg_Pt_^−1^, and a specific surface area activity equal to 3.97 mA cm_Pt_^−2^.

**Figure 23:**
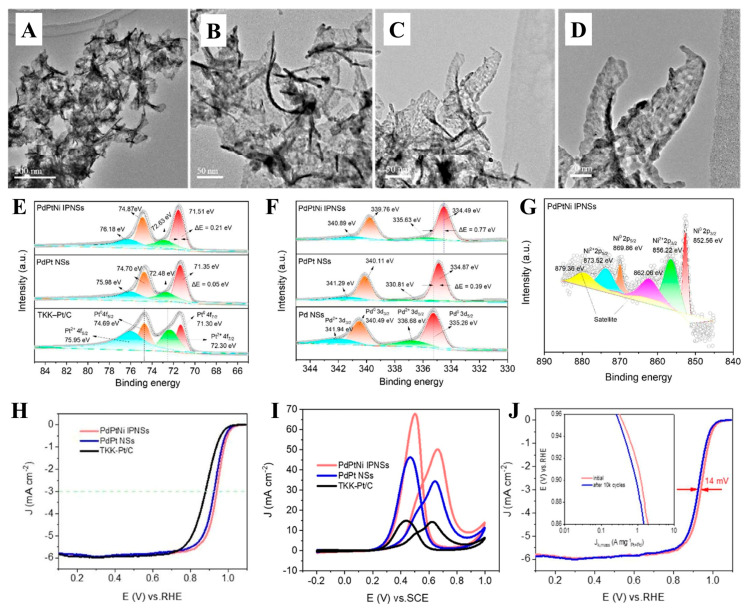
(**A**–**D**) The TEM characterization of Pd/Pt/Ni-IPNSs. (**E**) Pt 4f XPS spectra: Pd/Pt/Ni-IPNSs vs. Pd/Pt NSs vs. TKK-Pt/C. (**F**) Pd 3d XPS spectra: Pd/Pt/Ni-IPNSs vs. Pd/Pt-NSs vs. Pd-NSs. (**G**) Ni 2p XPS spectra of Pd/Pt/Ni-IPNSs. Electrochemical performance: (**H**) polarization curves. (**I**) CA curves at CP = 0.5 V. (*J*) Mass activity (Pt + Pd normalized): Polarization curves (main); TS values (inset); pre-/post-10,000 cycle comparison. Reprinted with permission from Ref. [108]. Copyright © 2023 Hydrogen Energy Publications LLC, Published by Elsevier Ltd. All rights reserved.

A Pd nanosheet with a smooth surface structure was synthesized by Chen et al. [108] via a solvothermal approach, and the Pd-Pt-Ni island particle nanosheet (Pd/Pt/Ni-IPNS) catalyst was further prepared using the deposition method. The prepared Pd/Pt/Ni-IPNSs exhibited excellent catalytic performance in ORR, MOR, and the ethanol oxidation reaction (EOR (Figure 23). Specifically, the ORR *E*_1/2_ value of Pd/Pt/Ni-IPNSs reached 0.939 V (vs. RHE), and the mass activity was 4.58 times greater compared to that of the commercial TKK-Pt/C catalyst. In EOR, the highest value of *J* was 2.06 times higher in comparison to that of the commercial Pt/C catalyst. In MOR, the highest value of *J* was 3.47 times higher compared to that of commercial Pt/C catalysts. By introducing Ni and Pt elements to produce a ternary alloy with Pd, the electronic structure of Pt was changed, and a synergistic impact was made. The Ni-induced helical structure facilitated the exposure of active atomic sites on the surface of the Pd/Pt/Ni-IPNSs catalyst, improved the ECSA and specific catalyst activity, and significantly improved the electrocatalytic efficiency of the catalyst.

Wang’s [109] team constructed a low platinum loading Pt/Cu/Co multi-component nanoalloy catalyst using the solvothermal method and systematically evaluated its multi-functional electrocatalytic performance for ORR and alcohol oxidation (MOR/EOR) in acid/base dual media. The material exhibited excellent full pH adaptability: the acidic ORR *E*_1/2_ value reached 0.948 V vs. RHE, and the mass activity was equal to 2.15 A mg^−1^ Pt, which was 13.3 times higher compared to that of the commercial Pt/C catalyst. In alkaline environments, the *E*_1/2_ value was 0.926 V vs. RHE, and the mass activity was 1.39 A mg^−1^ Pt, which was 10.7 times greater compared to that of the Pt/C catalyst. However, two critical challenges persist in this system: (1) heterogeneous distribution induces localized site deactivation through metal leaching; (2) inconsistent alkaline performance reveals insufficient electrolyte–catalyst interface optimization. In the future, the full pH stability and anti-poisoning ability can be synergistically improved by fine-tuning the spatial arrangement of alloy components and introducing interface stress control strategies.

**Table 7:** Summarized electrocatalytic activity of noble metal MACs.

Catalyst	HER Activity	Mass Activity	ORR Activity	Stability	Ref
Pt/Ru/Co TAs	TS = 26.2 mV/dec * η * = 15 mV (at *J* = 10 mA/cm^2^)	MA = 32.9 A/mg (at *η* = 50 mV)	NA	* η * increased 1 mV (at *J* = 10 mA/cm^2^, 20 h test)	[110]
N−Pt/HEA/C	NA	MA = 1.34 A/mg Pt (At 0.9 V) SA = 1.93 mA/cm^2^ (at 0.9 V)	*E*_1/2_ = 924 mV	After 30,000 cycles in ADT: MA decreased 20.9%, SA decreased 16.6%, and *E*_1/2_ decreased 8 mV After 30,000 accelerated stability test (AST) cycles in MEA, *J* decreased by 12.3% (at 0.7 V), ECSA decreased by 9.8%	[111]
Ir/Co/Ni-PHNCs	TS = 26.6 mV dec^−1^ *η *= 35 mV (at *J* = 20 mA/cm^2^)	NA	TS = 3.8 mV dec^−1^ *η *= 303 mV (at *J* = 10 mA/cm^2^)	*J* decreased by 5.5% (1.65 V vs. RHE) The polarization curve offset was 8 mV (1000 cycles durability test)	[112]
Pt_34_/Fe_5_/Ni_20_/Cu_31_/Mo_9_/Ru	TS = 27 mV dec^−1^ *η *= 20 mV (1 M KOH)	MA = 11.4 A mg^−1^	TS = 69 mV dec^−1^	In 1 M KOH with 40 h of the constant current test, *J* decreased by 5% (HER) and the current density retention rate = 92% (OER). In 0.1 M HClO_4_ with 40 h of the constant current test, the current density retention rate = 89% (ORR)	[113]
Fe/Ni/Co/Cr/Ru-HEA NPs	TS = 52.2 mV dec^−1^ *η* = 0.002 V (1 M KOH; *η* = 10 mA cm^−2^)	MA = 474.39 A g^−1^ (*η *= 0.1 V)	TS = 35 mV dec^−1^ *η *= 0.321 V (at *J* = 100 mA cm^−2^)	ΔV ≈ 0 (*J* = 250 mA cm^−2^, over the 3000 h cycle test)	[114]

Here, NA denotes non-availability; ADT denotes accelerated durability test; and SA denotes specific activity.

### 4.2. Non-Noble Metal MACs

Non-noble MACs leverage multi-metal coordination engineering to achieve the atomic-level modulation of active sites, combining cost-effectiveness, exceptional atomic economy, and earth-abundant metal compositions. These systems exhibit superior activity and selectivity through synergistic orbital hybridization and optimized intermediate adsorption/desorption energetics (Table 8).

Based on the high-entropy engineering strategy, Jian’s team successfully constructed a five-metal (Ce/Mn/Fe/Co/Ni) co-doped nitrogen–sulfur co-modified carbon-based catalyst (Ce/Mn/Fe/Co/Ni-NSC) through high-temperature pyrolysis. The material exhibited a two-dimensional porous layered structure, and its specific surface area was about 2.3 times higher in comparison to that of the single metal doping system (Fe-NC), exposing a rich, high-entropy active interface. The Ce/Mn/Fe/Co/Ni-NSCs catalyst demonstrated exceptional performance in direct liquid fuel cells, achieving peak power densities of 19.86 mW cm^−2^ for methanol oxidation (3.2 × higher than Fe-NC) and 73.81 mW cm^−2^ for borohydride oxidation. This enhancement verified the mechanism of the high-entropy multi-atomic synergistic effect to improve catalytic efficiency by optimizing the adsorption kinetics of intermediates.

**Figure 24:**
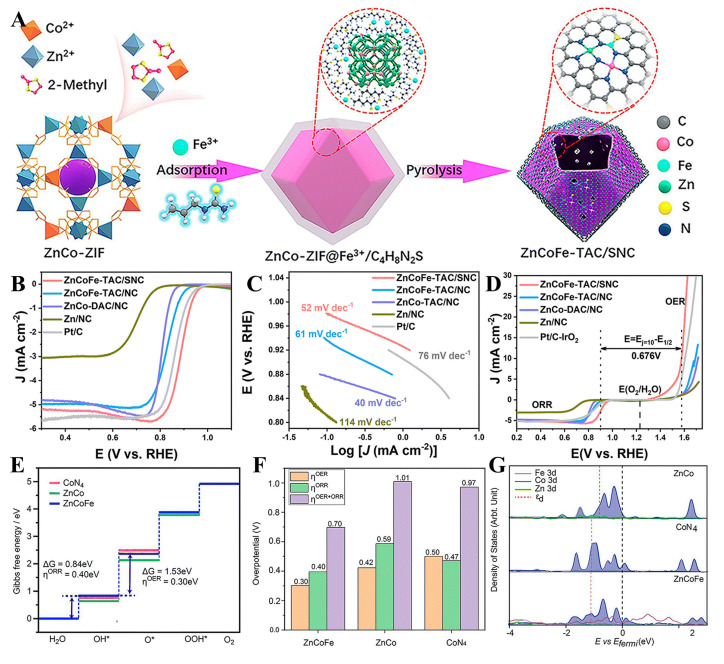
Synthesis and electrocatalytic performance. (**A**) A synthesis schematic of Zn/Co/Fe-TAC/SNC. (**B**) ORR/OER/Zn–air batteries. The obtained LSV profiles (Zn/Co/Fe-TAC/SNC and references). (**C**) The corresponding TS values. (**D**) Combined ORR/OER polarization plots. Theoretical calculations: (**E**) Free-energy diagrams (Zn/Co/Fe vs. CoN_4_ vs. Co/Zn). (Pink, blue, and green crosses = Zn/Co/Fe, CoN_4_, and Co/Zn.) (**F**) A histogram of *η* [orange (OER), green (ORR), purple (OER + ORR)]. (**G**) Diagram of the PDOS. (Black dotted line = Fermi level; red dotted line = d-band center.). Reprinted with permission from Ref. [115]. Copyright © The Royal Society of Chemistry 2024.

Cheng’s [115] team innovatively constructed a nitrogen/sulfur bi-coordinated Zn/Co/Fe hetero-trimetallic atom site catalyst (ZnN_3_/CoN_3_/FeN_2_/S @ SNC) through a two-step wet chemical strategy combining MOF topology construction and coordination engineering (Figure 24). The Zn/Co/Fe trimetallic catalyst demonstrated record-breaking bifunctional oxygen electrocatalysis in alkaline media, achieving an ORR *E*_1/2_ value equal to 0.901 V vs. RHE with a kinetic current density equaling 45.17 mA cm^−2^ at 0.85 V (2.3× Pt/C) while maintaining a low OER *η* value equal to 370 mV @10 mA cm^−2^ and an unprecedented Δ*E* equaling 0.676 V. When integrated into zinc–air batteries, it delivered a peak power density of 304 mW cm^−2^, specific capacity of 760 mAh g^−1^, and >500 h of cycling stability. Synchrotron XAS and DFT calculations revealed gradient 3d-orbital coupling among Zn/Co/Fe triatomic sites synergistically modulated by OOH adsorption configurations, enabling optimal four-electron pathway kinetics.

**Figure 25:**
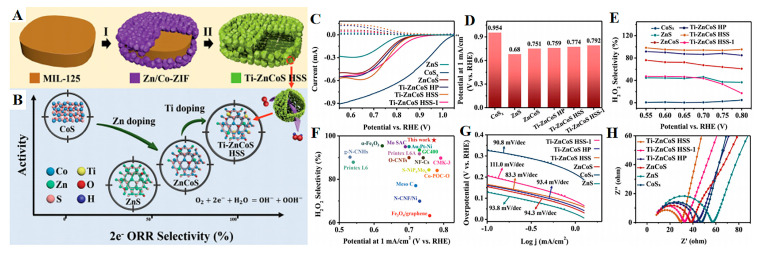
Synthesis and 2e^−^-ORR efficiency of Ti/Zn/Co/S-HSS. (**A**) The 2-step synthesis of Ti/Zn/Co/S-HSS. (**B**) 2e^−^-ORR activity/selectivity comparison: Co/S_x_, ZnS, Zn/Co/S, Ti/Zn/Co/S-HSS; electrochemical characterization (0.1 M KOH, O_2_-saturated). (**C**) The obtained LSV profiles: solid = disk current (I_d_); dashed = ring current (I_r_). (**D**) Onset potentials and (**E**) H_2_O_2_ selectivity of Co/S_x_, ZnS, Zn/Co/S, Ti/Zn/Co/S-HP, Ti/Zn/Co/S-HSS, and Ti/Zn/Co/S-HSS-1. (**F**) Benchmarking H_2_O_2_ activity/selectivity (at 0.55 V vs. RHE) vs. recently reported electrocatalysts. Kinetics and charge transfer: (**G**) TS values; (**H**) EIS Nyquist plots. Reprinted with permission from Ref. [116]. Copyright © 2022 The Authors. Advanced Science published by Wiley-VCH GmbH.

Zhang et al. [116] proposed a topological sulfidation strategy based on mixed MOFs and successfully constructed a Ti-doped Zn-Co sulfide hierarchical hollow superstructure catalyst (Ti/Zn/Co/S-HSS). The prepared catalyst demonstrated remarkable 2e^−^ ORR efficiency within alkaline environments, attaining an onset potential equal to 0.954 V vs. RHE (Figure 25D) with an *E*_1/2_ value at 0.774 V and an ultra-low TS value equal to 83.3 mV dec^−1^ (see Figure 25G). This was indicative of rapid reaction kinetics. It demonstrated 98% H_2_O_2_ selectivity and sustained production at 675 mmol h^−1^ g^−1^ with <5% activity decay over 50 h of continuous operation. The results showed that Ti doping-induced electronic structure reconstruction and multi-metal synergistic effects synergistically optimize the O_2_ adsorption configuration.

**Table 8:** Summarized electrocatalytic activity of non-noble metal MACs.

Catalyst	HER Activity	OER Activity	ORR Activity	Mass Activity	Stability	Ref
Zn/Co/Fe/TAC/SNC	NA	*η *= 360 mV (at *J* = 10 mA cm^−2^)	TS = 52 mV dec^−1^ E_1/2_ = 0.901 V (vs. RHE) *J* = 45.17 mA cm^−2^	NA	Att. ≈ 0 (0.1 M KOH, 5000 cycles stability test)	[115]
Co_x_/Ni-MAC	TS = 159.3 mV dec^−1^	NA	E_onset_ = 0.75 V (vs. RHE)	ECSA = 1.61 μF cm^−2^	Att. ≈ 0 (0.1 M HClO_4_, with constant disk potential of 0.5 V)	[117]
Co/Cr/Fe/Ni/Mo	TS = 46.09 mV dec^−1^ *η* = 156.7 mV (1.0 M KOH; *J* = 10 mA cm^−2^)	TS = 2.48 mV dec^−1^ * η * = 390 mV (1.0 M KOH; *J* = 50 mA cm^−2^.)	NA	NA	Δ*η* ≈ 0 (1.0 M KOH, 14 h test)	[118]
Fe/Co/Ni/Mn/V HEA/N-CNTs	NA	NA	E_onset_ = 0.99 V *E*_1/2_ = 0.85 V TS = 77.22 mV dec^−1^	NA	Δ*E*_1/2_ = 0 (10,000 cycles at 10 mV s^−1^ scan rate) *J* = 91.3% (10 h test at 0.6 V)	[119]
Mn/Se @ MWCNT	NA	TS = 54.76 mV dec^−1^ E_onset_ = 1.47 V *η* = 290 mV (at *J* = 10 mA cm^−2^)	E_onset_ = 0.94 V vs. RHE *E*_1/2_ = 0.86 V	NA	Att. = 4.7% (1.0 M KOH, 12 h test at 1.52 V) Att. = 9.8% (1.0 M KOH, 12 h test at 1.55 V)	[120]

Here, GCE denotes the glassy carbon electrode; E_onset_ denotes initial potential; NA denotes non-availability.

## 5. Summary and Future Prospects

The urgent transition to clean energy systems necessitates advanced electrocatalysts beyond conventional SACs. Recent advancements in noble/non-noble MACs demonstrate enhanced ORR/OER and HER activities through atomic-level synergistic effects. However, critical challenges hinder their practical deployment.

(1) Restricted synthesis methods. The conventional preparation routes of MACs generally rely on high-temperature calcination, which inherently lacks the precise regulation of the location of metal atoms on the support. This limitation obscures the identification of true active sites. Moreover, conventional pyrolysis inevitably induces metal aggregation (particle size > 5 nm) and energy inefficiency (>80% mass loss). Therefore, the development of novel synthesis strategies, which can achieve precise control over the configuration and location of multi-atom ensembles while simultaneously maintaining lower production costs, represents a key research direction in the future.
(2) Mechanistic ambiguity. Current characterization techniques for identifying MACs are limited to accurately distinguish MACs, making it difficult to precisely identify active sites and quantify their respective contributions to catalytic activity. Thus, the application of in situ techniques, such as in situ X-ray absorption spectroscopy (XAS) and in situ infrared spectroscopy (IR), is crucial for a deeper understanding of structure–performance relationships at the atomic level. Furthermore, in situ characterization can also monitor the dynamic evolution of active sites, which provides fundamental insights into the reaction process and clarifies the reaction mechanism.
(3) Traditional approaches generally entail prohibitive experimental labor due to the combinatorial complexity of MACs. The combinatorial optimization of atomic species/ratios exponentially escalates the experimental burden, severely hindering the development of MACs. Nowadays, machine learning can efficiently screen and predict high-performance MAC configurations, thereby drastically reducing experimental screening and accelerating their application in energy conversion and storage fields.

Therefore, future research should prioritize machine-learning-guided synthesis designs, earth-abundant MAC systems, and multimodal operando characterization to decipher structure–activity relationships while advancing roll-to-roll manufacturing technologies for industrial adoption.

## Figures and Tables

**Table 1:** ORR four-electron transfer process.

Four-Electron Transfer Process Under Acidic Conditions	Four-Electron Transfer Process Under Alkaline Conditions
* + O_2_ → O_2_*	* + O_2_ → O_2_*
O_2_* + H^+^ + e^−^ → OOH*	O_2_* + H_2_O + e^−^ → OOH* + OH^−^
OOH* + H^+^ + e^−^ → O* + H_2_O	OOH* + H_2_O + e^−^ → O* + OH^−^
O* + H^+^ + e^−^ → * + H_2_O	O* + H_2_O + e^−^ → * + OH^−^

* denotes the active site.

**Table 2:** ORR two-electron transfer process.

Two-Electron Transfer Process Under Acidic Conditions	Two-Electron Transfer Process Under Alkaline Conditions
* + O_2_ → O_2_*	* + O_2_ → O_2_*
O_2_* + H^+^ + e^−^ → OOH*	O_2_* + H_2_O + e^−^ → OOH* + OH^−^
OOH* + H^+^ + e^−^ → * + H_2_O_2_	OOH* + H_2_O + e^−^ → * + H_2_O_2_ + OH^−^

* denotes the active site.

**Table 3:** Expression of HER in different electrolytes.

The Elementary Reaction Steps of HER Under Alkaline Conditions	The Elementary Reaction Steps of HER in Acidic Conditions
H_2_O + e^−^ → OH^−^ + H_ads_ (Volmer)	H^+^ + e^−^ + * → H_ads_ (Volmer)
H_ads_ + H_2_O + e^−^ → OH^−^ + H_2_ (Heyrovsky)	H_ads_ + H^+^ + e^−^ → H_2_ (Heyrovsky)
Or 2H_ads_ → H_2_ (Tafel)	Or 2H_ads_ → H_2_ (Tafel)

Here, * denotes the active site on the surface of the catalyst, and ‘ads’ denotes the adsorption state of the intermediate product (H_ads_).

**Table 4:** Expression of OER in different electrolytes.

OER Under Acid Reaction Mechanism	OER Under Alkaline Reaction Mechanism
H_2_O(l) + * → *OH + H^+^ + e^−^	* + OH^−^ → *OH + e^−^
*OH → *O + H^+^ + e^−^	*OH + OH^−^ → *O + H_2_O(l) + e^−^
H_2_O(l) + *O → *OOH + H^+^ + e^−^	*O + OH^−^ → *OOH + e^−^
*OOH → * +O_2_(g) + H^+^ + e^−^	*OOH + OH^−^ → * + O_2_(g) + H_2_O(l) + e^−^

* denotes the active site.

## Data Availability

The supporting data used in this manuscript can be provided by the authors upon reasonable request.
